# An electrophoretic mobility shift assay with chemiluminescent readout to evaluate DNA-targeting oligonucleotide-based probes

**DOI:** 10.1371/journal.pone.0335674

**Published:** 2025-10-30

**Authors:** Michaela E. Everly, Peter J. Wieber, Ibrahim Al Janabi, Patrick J. Hrdlicka

**Affiliations:** Department of Chemistry, University of Idaho, Moscow, Idaho, United States of America; Purdue University, UNITED STATES OF AMERICA

## Abstract

A comprehensive and user-friendly method for evaluating recognition of double-stranded DNA (dsDNA) targets by oligonucleotide-based probes is presented. Thus, dsDNA-targeting probes such as single-stranded locked nucleic acids (LNAs) and double-stranded Invader probes are incubated with digoxigenin-labeled DNA hairpin targets, and the resulting recognition complexes are resolved using an electrophoretic mobility shift assay and tagged using a chemiluminescence immunoassay. Emissive products are detected by a C-DiGit Blot Scanner and quantified with the accompanying software. R-based scripts for data visualization and determination of *C*_50_ values (a measure of the dsDNA-binding affinity of a probe) are also provided. The data presented here demonstrate the effectiveness of the described protocol and highlight the variable dsDNA-recognition efficiencies of LNAs, Invader probes, and chimeric Invader:LNA probes.

## Introduction

Recent advancements in nucleic acid (NA) chemistry and biology have led to an accelerated development of NA-based therapeutics against genetic diseases. In fact, more than half of the approximately twenty FDA-approved NA-based therapeutics have gained approval within the past five years [1]. These include protein-targeting aptamers, RNA-targeting antisense oligonucleotides, small interfering RNAs, and other NA-based systems (i.e., mRNA vaccines and an *ex vivo* CRISPR-Cas9 gene therapy). In contrast, simple DNA-targeting oligonucleotides (ONs) and NA mimics have yet to reach the clinic as development of a robust and generally applicable probe technology has proven difficult. This is largely due to the additional pharmacodynamic and pharmacokinetic challenges posed by targeting chromosomal DNA, which include the need for compartment-specific delivery and limited access to nucleotide-specific molecular features to enable specific binding. Considerable efforts have been devoted to the development of chemically modified ON probes and NA mimics with increased affinity towards double-stranded (ds) DNA targets. These include different groove-binding approaches such as polyamides and triplex-forming oligonucleotides (TFOs) and peptide nucleic acids (PNAs), [2–5] as well as strand-invading strategies, i.e., probes capable of overcoming the existing Watson-Crick base pairs of dsDNA to form new, more stable Watson-Crick base pairs between probe strands and complementary DNA. Notable examples of the latter include modified single-stranded PNAs [6–11] and double-stranded pseudocomplementary PNAs, [12–18] among other approaches [19–21].

**Fig 1:**
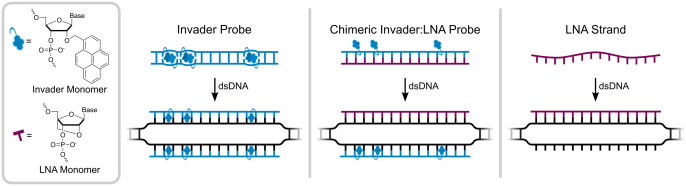
Structures of 2′-*O*-(pyren-1-yl)methyl-RNA (Invader) and locked nucleic acid (LNA) monomers, and illustrations of dsDNA-invasion mechanisms using Invader probes, chimeric Invader:LNA probes, and single-stranded LNAs.

Our group has pursued the development of Invader probes [22–26] for recognition of mixed-sequence dsDNA targets via double-duplex invasion. Invader probes are DNA duplexes with +1 interstrand zipper arrangements of intercalator-modified nucleotides such as 2′-*O*-(pyren-1-yl)methyl-RNA (Fig 1). This specific motif results in a perturbed and energetically activated probe duplex as it violates the nearest neighbor exclusion principle (NNEP) [27,28], which states that a local intercalator density exceeding one per two base pairs is unfavorable due to excessive unwinding of the DNA duplex and perturbed base-pair stacking. Meanwhile, the individual probe strands exhibit high affinity for complementary DNA (cDNA) as duplex formation results in strongly stabilizing stacking interactions between the intercalator and flanking base pairs (i.e., local intercalator density is at the NNEP limit). These stability differences provide the driving force for mixed-sequence dsDNA-recognition via a double-duplex invasion process that has been used to detect specific chromosomal DNA sequences [24–26].

As an extension of our original probe design, we recently introduced chimeric duplexes between individual Invader strands and fully modified locked nucleic acid (LNA) [29] strands, i.e., Invader:LNA probes [30]. Intercalators are generally poorly accommodated in *A*-type (RNA-like) duplexes (Fig 1) [31,32]. As a result, the double-stranded Invader:LNA probes are also energetically activated for dsDNA-recognition, as the individual Invader and LNA strands exhibit high cDNA affinity, while the chimeric probes are more labile. The data presented herein were generated, following the described protocol, during our characterization of additional chimeric Invader:LNA probes.

**Fig 2:**
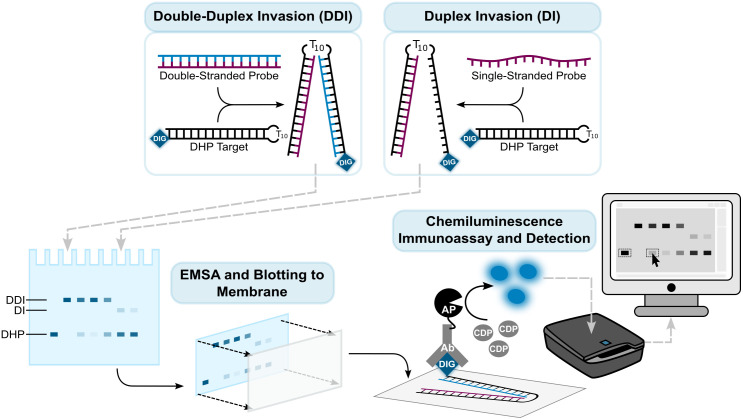
Overview of the protocol described herein.

A key step in the development of strand-invading ON probes is to evaluate their affinity towards complementary dsDNA targets. One way this can be accomplished, is to couple an invasion assay (probe + model dsDNA target) with an electrophoretic mobility shift assay (EMSA) that utilizes a suitably sensitive signal-generating readout. EMSAs are used extensively for purification of nucleic acids and to study interactions between biomolecules, and the literature is replete with protocols for such applications. Detection of relevant NA-based species within acrylamide gels is typically realized using non-specific dyes (e.g., ethidium bromide or SYBR Gold), or end-labeling of ONs with fluorophores (e.g., FAM or Cy5) or radioactive species (e.g., ^32^P). While non-specific dyes offer a low-cost approach, they are limited in sensitivity and are not suitable if a large probe excess is used, since the signal from the unbound probe will be very intense. Fluorescent and radioactive methods provide high sensitivity but require specialized equipment and can have high associated costs. In addition, radiolabeling requires specialized training and dedicated laboratory space to minimize risks due to radioactivity. These readouts were not available or feasible for our specific applications, which led us to develop the present EMSA with chemiluminescent readout to determine the binding affinity of DNA-targeting ON-based probes. While the assay has been described in general terms in the experimental sections of prior peer-reviewed research articles published by our group [30], we here provide a comprehensive protocol to evaluate duplex or double-duplex invasion of model dsDNA targets by representative ON probes such as single-stranded LNAs, double-stranded Invader probes or double-stranded chimeric Invader:LNA probes (Fig 2). The detailed step-by-step protocol will eliminate a hurdle for prospective researchers wishing to enter this field of research. The protocol combines an array of established techniques and is comprised of the following subprotocols: (i) Invasion Assay, (ii) EMSA and Blotting, (iii) Chemiluminescence Immunoassay, (iv) Signal Detection and Quantification, (v) Dose-Response Invasion Assay, (vi) Data Visualization and *C*_50_ Calculations with R, and (vii) Suggestions for Increasing Throughput.

Our laboratory has been using digoxigenin (DIG)-labeled DNA hairpins (DHPs) as convenient model dsDNA targets for preliminary evaluation of dsDNA-binding characteristics of various single- and double-stranded probes. The DHP is comprised of a double-stranded region that is complementary to the probes and linked on one end via a T_10_ loop, resulting in a high-melting hairpin. Other model dsDNA targets and assays have been considered including:

(i) *Complementary DNA duplexes* (equivalent to the DHP targets lacking the connecting single-stranded T_10_ region). These targets were used in our earliest studies and coupled with a fluorometric readout (monitoring the disappearance of a pyrene excimer signal present in intact Invader probes) [22,23] or with EMSAs and SYBR gold staining [23]. While very convenient, these targets were superseded by the DHP targets over concerns for partial target denaturation. DHPs are more stable than DNA duplexes due to their unimolecular nature, which renders end-fraying less likely and provides a model target that is more representative of chromosomal DNA.
(ii) *PCR fragments with complementary internal target regions.* These targets were not utilized over concerns for minimal mobility differences between unbound targets and recognition complexes in EMSAs and since ^32^P-labeling was not available to us. Along similar lines, we could not use such targets in isothermal calorimetry or surface plasmon resonance based binding affinity assays due to a lack of access to the necessary instrumentation.
(iii) *Chromosomal DNA targets coupled with fluorescence in situ hybridization (FISH).* We have successfully evaluated various Invader probes using such assays [24–26]. However, this target type and assay require culturing of cells, isolation of chromosomal DNA with a suitable high copy-number target region to generate sufficient signal, fluorophore-labelling of the DNA-targeting probe, and access to a fluorescence microscope. Moreover, this assay provides a qualitative, not quantitative, assessment of the dsDNA-binding properties of the probes.

It is for these reasons that we use DIG-labeled DHPs in combination with an EMSA with chemiluminescent readout to evaluate DNA-targeting oligonucleotide-based probes.

In the present approach, double- or single-stranded probes are incubated with digoxigenin-labeled DHP targets and expected to form recognition complexes via double-duplex invasion (DDI) or duplex invasion (DI), respectively (Fig 2). The resulting recognition complexes are resolved in an EMSA and subsequently blotted onto positively charged nylon membranes. Membrane-bound DHPs and recognition complexes are then treated with alkaline phosphatase (AP)-conjugated anti-DIG antibody (Ab) and CPD-Star (or CSPD) substrate in a chemiluminescence immunoassay, in which emissive products are detected via a C-DiGit Blot Scanner and quantified with Image Studio software.

## Materials and methods

The protocol described in this peer-reviewed article is published on protocols.io, https://dx.doi.org/10.17504/protocols.io.4r3l218rxg1y/v1, and is included for printing as S1 File with this article.

### Probe selection

Fourteen ONs were evaluated as single-stranded probes, as well as being used to construct ten double-stranded probes (four Invader probes and six chimeric Invader:LNA probes) (Table 2 in the S3 File). The Invader probes were available from a prior study and originally designed to be complementary to specific dsDNA sequences in common food-borne pathogens, i.e., *E. coli* (two different regions, herein referred to as *E. coli* (R1) and *E. coli* (R2)), *S. enterica*, and *C. jejuni* [33]. Some of the Invader probe strands used herein have 3’-biotin or 5’-amino-modifier modifications, which were needed for the prior study but are not necessary nor utilized in the current protocol. Thus, a total of 24 pathogen-specific probes were evaluated for their biophysical properties (Table 2 in the S3 File) and subjected to the described protocol to evaluate their dsDNA-binding characteristics against complementary DIG-DHP targets (**DH1‒DH4**).

### Probe quantification

Probe concentrations were determined using a Cary 100 UV/VIS spectrophotometer, equipped with a 12-cell Peltier temperature controller, and using quartz optical cells with a path length of 1.0 cm. Concentrations were estimated by recording *A*_260_ values for individual strands and using the following extinction coefficients for DNA and pyrene (OD_260_/μmol: G (12.01), A (15.20), T (8.40), C (7.05), and pyrene (22.4) [34]) or RNA (OD_260_/μmol: G (13.7), A (15.4), T (10.0), and ^5-Me^C (9.0)) for Invader or LNA strands, respectively.

### DNA hairpin invasion assays

14-mer DHPs (**DH1**: 5’-AACAGTTCTATCAG-T_10_-CTGATAGAACTGTT-3’; **DH2**: 5’-GCATGGCTCTTGAT-T_10_-ATCAAGAGCCATGC-3’; **DH3**: 5’-TCGTTATTGGCGAT-T_10_-ATCGCCAATAACGA-3’; **DH4**: 5’-TATGCCATTTGAAA-T_10_-TTTCAAATGGCATA-3’), were obtained from commercial sources and used without further purification. DHPs were DIG-labeled and incubated with complementary single- or double-stranded probes (at 25-fold molar probe excess for the initial Invasion Assay or variable probe concentration for the Dose-Response Invasion Assay) as described in the protocol.

### EMSA, blotting, and chemiluminescence immunoassay

After the Invasion Assay, recognition complexes and unbound DHPs were resolved in 20% non-denaturing polyacrylamide gels via electrophoresis (~4 h) and subsequently blotted onto positively charged nylon membranes as described in the protocol. The chemiluminescence immunoassay and signal detection were also completed as described, using CDP-Star as the substrate.

### Data representation and *C*_50_ calculations

An R script (see *Associated Content*) was written to fit data points from dose-response experiments to the following equation: *y* = *C* + *A* (1 − *e*^−*kt*^) where *C*, *A* and *k* are fitting constants, using the Levenberg-Marquardt nonlinear least-squares algorithm from the MINPACK library in R [35]. The R script then used the resulting equation to calculate *C*_50_ values by setting *y* = 50 and solving for *t*. The same script was used to construct the dose-response graph. All images were assembled in Inkscape.

## Results

**Fig 3:**
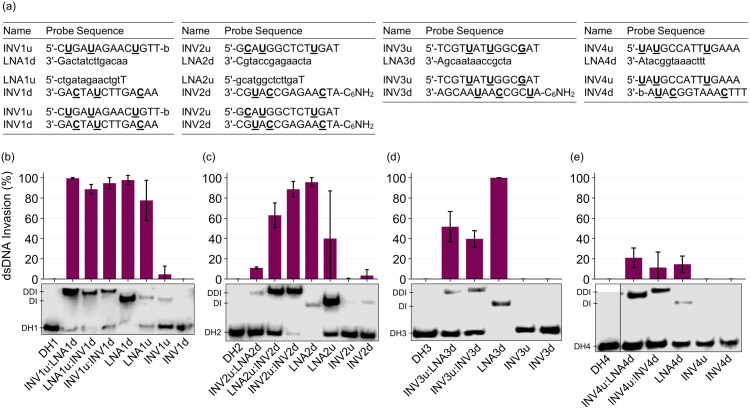
Example data from the Invasion Assay with various single- and double-stranded ON probes. **(a)** Sequences of probes used herein and (b-e) representative Invasion Assay results using the described protocol. Histograms represent the mean percent invasion of the complementary DHP (**DH1‒DH4**); error bars represent the standard deviation from at least three trials. As detailed in the protocol, probes were incubated with complementary DIG-DHP targets at a 25-fold molar probe excess for 17 h at 37 °C in Invasion Buffer (50 mM HEPES, 100 mM NaCl, 5 mM MgCl_2_, pH 7.2, 10% sucrose, 1.44 mM spermine tetrahydrochloride), subsequently resolved in 20% non-denaturing polyacrylamide gels, and detected using CDP-Star as the chemiluminescence substrate. Underlined residues = 2′-*O*-(pyren-1-yl)methyluridine monomers; lower case residues = LNA monomers; “b” = 3’-biotin TEG; C_6_NH_2_ = 5’-amino-modifier C6; “u” and “d” in probe names correspond to “up” and “down”, respectively; DDI = double-duplex invasion recognition complex; DI = duplex invasion recognition complex. The leftmost lane in each electrophoretogram is the indicated DHP without probe. The electrophoretogram in (d) is a composite of two separate gel images. See the S2 File for uncropped blot images. Note, the 3’-biotin TEG and 5’-amino-modifier modifications present in some Invader probes are not necessary for the protocol.

The invasion efficiency of 24 pathogen-specific probes, i.e., fourteen single-stranded probes and ten double-stranded probes (Fig 3a) was evaluated using the described protocol. The probes – initially used at 25-fold molar excess – were incubated with complementary DIG-labeled DHP targets at 37 °C for 17 h. The resulting incubation mixtures were resolved in 20% non-denaturing polyacrylamide gels, transferred to positively charged nylon membranes, and permanently fixed to the membranes via UV-induced formation of covalent linkages between the nitrogenous bases of the ONs and amines on the membrane surface. Visualization of the membrane-fixed DIG-DHP species (unbound or bound by the probes) was realized using an immunological chemiluminescence assay (Fig 2). In short, membranes were treated with alkaline phosphatase (AP)-conjugated anti-DIG antibody F_ab_ fragments that specifically bind to DIG-labeled DHP species. Upon addition of an AP-specific chemiluminescence substrate (e.g., CSPD or CDP-Star), the substrate is dephosphorylated by AP resulting in an unstable intermediate that rapidly decomposes to an excited benzoate species [36]. As the excited species decays to its ground state, light is emitted and then captured by a LI-COR C-DiGit Blot Scanner. The C-DiGit Blot Scanner is equipped with a charge-coupled device (CCD) that converts emitted photons to quantifiable electrical signals. The bound or unbound DIG-DHP species appear as “bands” in the generated image (e.g., see electrophoretograms in Fig 3b–e). The signal intensity of each band is correlated to the number of photons emitted from the DIG-DHP species, which in turn is correlated to the concentration of the specific DIG-DHP species (Fig 10 in the S3 File). The pixels appearing within a band were quantified, and the invasion efficiency of a probe was calculated as the ratio of signal intensity between the bound and unbound DIG-DHP species.

Recognition of DIG-labeled DHP-targets by single-stranded or double-stranded probes results in the formation of invasion complexes with a greater hydrodynamic radius than the unbound DIG-DHP. Thus, unbound DIG-DHPs display the greatest mobility, followed by duplex invasion (DI) complexes and double-duplex invasion (DDI) complexes (e.g., note relative band positions for DHP, DI, and DDI complexes in Fig 3b–e). Some of the single-stranded probes evaluated here resulted in substantial (i.e., > 50%) recognition of the corresponding DHP targets (i.e., **LNA1d**, **LNA1u**, **LNA2d**, and **LNA3d**), whereas others led to little recognition (i.e., **LNA2u**, **LNA4d**, and all individual Invader strands). Similarly, some double-stranded probes resulted in substantial recognition (i.e., **INV1u**:**LNA1d**, **LNA1u**:**INV1d**, **INV1u**:**INV1d**, **LNA2u**:**INV2d**, **INV2u**:**INV2d**, and **INV3u**:**LNA3d**), while others did not (i.e., **INV2u**:**LNA2d**, **INV3u**:**INV3d**, **INV4u**:**LNA4d**, and **INV4u**:**INV4d**). For a detailed discussion of the biophysical properties and dsDNA-recognition characteristics of the different LNA, Invader and chimeric Invader:LNA probes, see the S3 File.

**Fig 4:**
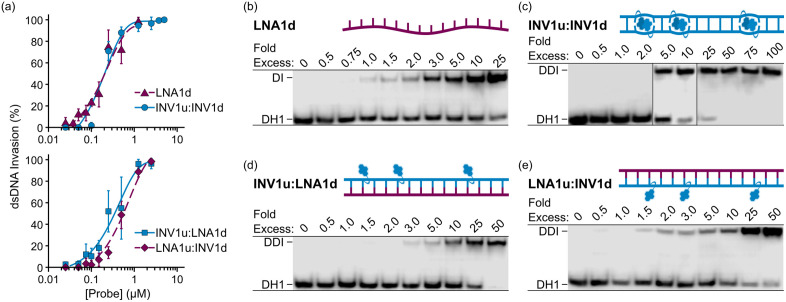
Representative results from Dose-Response Invasion Assays. **(a)** Dose-response curves were generated with data obtained from dose-response experiments and plotted using the Dose-Response R Script (see *Associated Content*). **(b‒e)** Representative electrophoretograms from dose-response experiments for recognition of **DH1** by the indicated single- or double-stranded probes. Error bars represent the standard deviation from at least three trials. DI = duplex invasion; DDI = double-duplex invasion. For experimental conditions, see Fig 3. The electrophoretogram in (c) is a composite of two separate gel images. See the S2 File for uncropped blot images.

Dose-response assays, where probe concentrations are incrementally increased, can be used to determine the probe concentration that results in 50% recognition of a target (i.e., the *C*_50_ value), which is a quantitative measure of a probe’s dsDNA-affinity. Fig 4 shows the data generated from dose-response experiments using a selection of single- and double-stranded probes. The calculated *C*_50_ values are shown in Table 1.

**Table 1:** *C*_50_ values and percent invasion of DH1 by various single-stranded and double-stranded probes^*a*^.

Probe	*C*_50_ (μM)	Invasion at 25x (%)
**LNA1d**	0.20	98 ± 5
**INV1u**:**INV1d**	0.20	95 ± 6
**INV1u**:**LNA1d**	0.35	96 ± 4
**LNA1u**:**INV1d**	0.55	89 ± 5

^*a*^Calculated from the dose-response curves shown in Fig 4a using the companion R script (see *Associated Content*). “Invasion at 25x” = percent of **DH1** invasion when using a 25-fold molar probe excess. ± = standard deviation from at least three trials.

**LNA1d**, **INV1u**:**LNA1d**, and **LNA1u:INV1d** exemplify the results expected for dose-response assays: as the probe concentration increases, the signal intensity of recognition bands gradually increases along with a corresponding decrease in unbound DHP signal intensity (Figs 4b, 4d, and 4e). In contrast, **INV1u**:**INV1d** results in less than 2% recognition at 2-fold molar probe excess but ~70% recognition at 5-fold molar probe excess (Fig 4c). This is not ideal, as the function used to generate the fitted curve, from which the *C*_50_ is calculated, will be less accurate due to the lack of data points near the predicted *C*_50_. This can be rectified by inclusion of additional data points from experiments where between 2- and 5-fold molar excess of **INV1u**:**INV1d** is used.

## Conclusion

The protocol described herein provides a comprehensive, reasonably cost-effective, and user-friendly method for evaluating duplex and double-duplex invasion of model dsDNA targets using single-stranded probes like LNAs or γPNAs, and double-stranded probes like Invader probes, chimeric Invader:LNA or Invader:γPNA [30,37,38]. The representative data provided here demonstrate the effectiveness of the described protocol while highlighting the variability in recognition efficiency of model dsDNA targets by such probes. Given its robustness and adaptability, this protocol is expected to be applicable towards many types of DNA-targeting probes.

## Supporting information

S1 File Step-by-step protocol, also available on protocols.io (https://doi.org//10.17504/protocols.io.4r3l218rxg1y/v1). (PDF)

S2 File Raw images. (PDF)

S3 File Supporting data. (DOCX)
