# Supplementary material for: An electrophoretic mobility shift assay with chemiluminescent readout to evaluate DNA-targeting oligonucleotide-based probes
Source: PLoS One. 2025 Oct 30;20(10):e0335674. doi: 10.1371/journal.pone.0335674 (PMC12574872; doi:10.1371/journal.pone.0335674)
Supplement: S1 File — (PDF) [file pone.0335674.s001.pdf]

Jul 29, 2025

An electrophoretic mobility shift assay with chemiluminescent readout to evaluate DNA-targeting oligonucleotide-based probes

DOI
dx.doi.org/10.17504/protocols.io.4r3l218rxg1y/v1

Michaela E. Everly[1], Peter J. Wieber[1], Ibrahim Al Janabi[1], Patrick J. Hrdlicka[1]

[1]Department of Chemistry, University of Idaho, Moscow, Idaho 83844-2343, USA

Patrick J. Hrdlicka: Corresponding author

Michaela protocol

Michaela E. Everly
University of Idaho

OPEN ACCESS

DOI: dx.doi.org/10.17504/protocols.io.4r3l218rxg1y/v1

Protocol Citation: Michaela E. Everly, Peter J. Wieber, Ibrahim Al Janabi, Patrick J. Hrdlicka 2025. An electrophoretic mobility shift assay with chemiluminescent readout to evaluate DNA-targeting oligonucleotide-based probes. **protocols.io** https://dx.doi.org/10.17504/protocols.io.4r3l218rxg1y/v1

License: This is an open access protocol distributed under the terms of the **Creative Commons Attribution License**, which permits unrestricted use, distribution, and reproduction in any medium, provided the original author and source are credited

Protocol status: Working
We use this protocol and it's working

Created: June 30, 2025

Last Modified: July 29, 2025

Protocol Integer ID: 222791

Keywords: DNA recognition, Duplex invasion, Double-duplex invasion, Electrophoretic mobility shift assay, C-DiGit, CSPD, CDP-Star, Locked nucleic acid (LNA), Invader probes, Antigene, Oligonucleotides, Intercalation, Pyrene

Funders Acknowledgements:
Institutional Development Award (IDeA) from the National Institute of General Medical Sciences of the National Institutes of Health
Grant ID: P20GM103408

Abstract

A comprehensive and user-friendly method for evaluating recognition of double-stranded DNA (dsDNA) targets by oligonucleotide-based probes is presented. Thus, dsDNA-targeting probes such as single-stranded locked nucleic acids (LNAs) and double-stranded Invader probes are incubated with digoxigenin-labeled DNA hairpin targets, and the resulting recognition complexes are resolved in an electrophoretic mobility shift assay and tagged using a chemiluminescence immunoassay. Emissive products are detected by a C-DiGit Blot Scanner and quantified with the accompanying software. R-based scripts for data visualization and determination of C_{50} values (a measure of the dsDNA-binding affinity of a probe) are also provided. The data presented here demonstrate the effectiveness of the described protocol and highlight the variable dsDNA-recognition efficiencies of LNAs, Invader probes, and chimeric Invader:LNA probes.

Attachments

XLSX

Invasion Assay Gel I...

26KB

Guidelines

COMMONLY USED ABBREVIATIONS:

- DHP = DNA hairpin
- diH_2O = deionized water
- EMSA = electrophoretic mobility shift assay
- MCT = microcentrifuge tube
- ON = oligonucleotide
- RT = room temperature

RECIPE GUIDELINES:

- Prepare all solutions and buffers diH_2O, then sterilize as indicated in the recipes listed under the *Materials* tab. Unless otherwise noted, make all solutions prior to starting the protocol.
- Solutions/buffers that are not sterilized and used immediately should be prepared using autoclaved diH_2O or double-distilled water and portioned into smaller containers to reduce the likelihood of contamination.
- Use a stir plate or vortex mixer at RT to completely dissolve solid materials for solutions/buffers. Do not use heat unless specified in the recipe notes.
- Each recipe includes a recommended shelf life. This should be considered as the maximum period a solution/buffer can be stored, assuming proper storage requirements are met and the solution remains contaminant-free. Routinely check solutions and buffers for precipitation, bacterial/mold growth, and other contaminants. In some instances, precipitates may be redissolved through heating or filtered off (indicated in recipe notes). Solutions/buffers with bacterial or mold growth must be discarded. Routinely check the pH of solutions/buffers if pH is vital to the experiment or if the solution is susceptible to pH changes. Solutions should be remade if substantial pH changes are observed.
- This protocol is sectioned into seven parts: (i) Invasion Assay, (ii) EMSA and Blotting, (iii) Chemiluminescence Immunoassay, (iv) Signal Detection and Quantification, (v) Dose-Response Invasion Assay, (vi) Data Visualization and C_{50} Calculations with R, and (vii) Suggestions for Increasing Throughput. Read the entire protocol prior to start. The entire protocol will take approximately 25–28 h, depending on the substrate used for immunological detection (Table 1). Suitable stopping points are indicated in the protocol.

ADDITIONAL NOTES:

- Throughout the protocol, the term “probe” is used to describe both single-stranded and double-stranded dsDNA-targeting ONs or nucleic acid mimics (e.g., single-stranded LNA or γPNA probes, and double-stranded Invader probes, chimeric Invader:LNA probes, or chimeric Invader:γPNA probes).
- Membranes should be handled gently using forceps along the edges of the membrane.

- Volumes of membrane processing solutions are dependent on the size of the membrane being processed (see Table 4). To simplify the protocol, we suggest cutting membranes to the same size for every EMSA gel (as instructed in Step 13). While the results from the first successfully completed gel can be used as a reference, subsequent gels should still be measured to ensure the membrane is properly sized. Once several gels have been recorded and dimensions are well-established, membrane processing solutions can be pre-aliquoted to speed-up operations.

A	B	C	D	E	F
**1x Maleic Acid Buffer**	**1x Washing Buffer**	**Detection Buffer**	**1x Blocking Solution**	**Antibody Working Solution**	**Substrate Working Solution**
1 mL/cm^2 membrane	1 mL/cm^2 membrane	1 mL/cm^2membrane	1 mL/cm^2 membrane	1 µL per 10 mL Blocking Solution (75 mU/mL)	0.03 mL/cm^2 membrane.

Table 4. Buffer and solution volumes for the Chemiluminescence Immunoassay.

- The CSPD substrate reaches peak light emission in 3–4 h, which can persist for days **[5]**. We found that an initial incubation at 37 °C for 10 min, followed by 3–5 h at RT (shielded from light, completely sealed in hybridization jacket), resulted in sufficient chemiluminescence signal to be captured by the C-DiGit Blot Scanner. Incubation times shorter than 3 h resulted in variable and/or weak band intensities, suggesting the chemiluminescent signal output from CSPD was near the detection limit of the scanner. When additional captures were taken of the same blot, a reduction in band intensity – and in some cases complete disappearance of bands – was observed, even when the blot was returned to the hybridization jacket between captures. Presumably, this is due to the chemiluminescence reaction slowing as the blot begins to dry. Accordingly, we recommend avoiding multiple captures of blots treated with the CSPD substrate. If additional captures are necessary, the blot can be imaged without removing it from the hybridization jacket. However, this may lead to increased background signals if the plastic is not completely smooth on the scanner surface, and bands may appear “fuzzy” due to signal diffusion by the substrate solution in the hybridization jacket.
- The CDP-Star substrate produces a faster and more intense signal than CSPD, with peak light emission after 1–2 h (~10-fold brighter signal) **[5]**. Sufficient signal for capture by the C-DiGit Blot Scanner is produced after 10 min incubation at 37 °C and the signal was found to persist with minimal loss in intensity 24 h after initial capture (blot was returned to and sealed in the hybridization jacket between captures). Thus, multiple captures can be taken if the CDP-Star substrate is used. Due to its fast reaction rate and much higher signal intensity, we prefer CDP-Star as the substrate. However, CSPD is less expensive.
- Many other protocols, manuals, and product information sheets discussing construction of hybridization jackets (with CSPD, CDP-Star, or other substrates) specify that blots should not be covered with plastic wrap but instead be covered with acetate sheet protectors, hybridization bags, or transparency films. We have consistently used food-grade plastic wrap to cover the blots and have not encountered any problems.

Materials

General Equipment:

A	B
**Equipment**	**Vendor (Model)/Catalogue Number**
Micropipettes	Gilson, Accura, Rainin Pipet-lite (P10, P20, P200, P1000)
0.5 and 1.5 mL microcentrifuge tubes	VWR/76332-066 and -068
15 mL centrifuge tubes	VWR/89039-664
50 mL centrifuge tubes	VWR/470225-002
Dual-range analytical balance	Mettler Toledo (XS105)
Autoclave	Market Forge (Sterilmatic)
Stirring hot plate	Fisher Scientific (Isotemp)
pH meter	Denver Instrument (UB-10)
Oven (37 °C)	Quincy Lab, Inc. (10-140E Incubator)
Heat block with appropriately sized inserts for 0.5 mL microcentrifuge tubes, set to 90 °C	VWR/75838-286
CentriVap Benchtop Centrifugal Vacuum Concentrator with scroll dry pump	LABCONCO/7810010; Edwards (XDS 5c)
Vortex mixer	Fisher Scientific/02215365
Mini centrifuge (for brief spins)	BioExpress (GeneMate C-1301-PC)
High-speed centrifuge (>10000 rpm)	Beckman Coulter (Microfuge 18)
Shaking/rotating platform	Labline (MAXI)
Bio-Rad gel casting system	Bio-Rad/1658052
Mini-PROTEAN® Tetra Cell, Mini Trans-Blot®, and casting system or mPAGE® Mini Gel and Transfer systems	Bio-Rad/1658029 or MilliporeSigma/MGT and MWTS
Power supply (for gel tanks)	VWR (AccuPower 300)
Mini-fridge or cold room (4 °C)	Various
UV crosslinker	Stratagene (Stratalinker 2400)
C-DiGit® Blot Scanner	LI-COR Biotech
Image Studio™ Version 5 (C-DiGit)	LI-COR Biotech
Computer to run Image Studio™	Various
Sealable plastic bags	Ziploc (sandwich)
Assay containers (e.g., pipette boxes/lids)	Various
Plastic forceps	Various
Plastic wrap	Kirkland (stretch-tite)
70% Aqueous ethanol, in spray bottles	Various

A	B
Test tube or roller (for removing air bubbles from blots)	Various
Filter paper (0.22 µm)	Various
Magnetic stir bars	Various
Stir rod	Various
Beakers (variable sizes)	Various
Lab-grade glass bottles for storing solutions/buffers (variable sizes)	Various
Lab-grade amber glass bottles (for storing acrylamide solutions)	Various
Lab-grade plastic bottles for storing acidic solutions/buffers (variable sizes)	Various
Graduated cylinders (variable sizes)	Various

Microcentrifuge Tubes **VWR International (Avantor) Catalog #**76332-066

15-mL conical tubes, sterile **VWR International (Avantor) Catalog #**89039-664

Centrifuge Tubes **VWR International (Avantor) Catalog #**470225-002

Balance **Mettler Toledo Catalog #**XS105

Mini-PROTEAN® Tetra Cell Casting Stand with Clamp Kit for Single Core System **Bio-Rad Laboratories Catalog #**1658052

Mini-PROTEAN® Tetra Cell and Mini Trans-Blot® Module **Bio-Rad Laboratories Catalog #**1658029

Invasion Assay (Steps 1–5 & 38–39):

A	B
**Materials**	**Vendor/Catalogue Number**
Bespoke DNA-targeting probes	Synthesized in-house (LNAs may be purchased from IDT)
DNA Hairpin Targets (model targets for bespoke probes)	IDT
Terminal Transferase from calf thymus, recombinant, E. coli (5x TdT Reaction Buffer and 25 mM CoCl2 solution included)	MilliporeSigma/3333566001
Digoxigenin-11-ddUTP	MilliporeSigma/11363905910
EDTA (disodium salt dihydrate, molecular biology grade, >99% purity)	MilliporeSigma/E5134-50G
HEPES (molecular biology grade, >99% purity)	VWR/97061-826
Sodium chloride (molecular biology grade, ≥99.8% purity)	VWR/TS32730-0010

A	B
Magnesium chloride, hexahydrate (molecular biology grade, ≥99% pure)	MilliporeSigma/442611-M
Sucrose (molecular biology grade, >99% purity)	MilliporeSigma/573113
Spermine tetrachloride (molecular biology grade)	MilliporeSigma/S1141

Terminal Transferase from Calf Thymus, recombinant, E. coli **Merck MilliporeSigma (Sigma-Aldrich) Catalog** #3333566001

Digoxigenin-11-ddUTP **Merck MilliporeSigma (Sigma-Aldrich) Catalog** #11363905910

EDTA disodium salt **Merck MilliporeSigma (Sigma-Aldrich) Catalog** #E5134-50G

Magnesium Chloride, Hexahydrate, Molecular Biology Grade **Merck MilliporeSigma (Sigma-Aldrich) Catalog** #442611-500GM

Sucrose **Merck MilliporeSigma (Sigma-Aldrich) Catalog** #573113

Spermine tetrahydrochloride **Merck MilliporeSigma (Sigma-Aldrich) Catalog** #S1141

EMSA and Blotting (Steps 6–20):

A	B
**Materials**	**Vendor/Catalogue Number**
Tris Base (molecular biology grade, ≥99.8% purity)	Fisher Scientific/BP152-500
Boric acid (molecular biology grade, ≥99.5% purity)	MilliporeSigma/B6768-500G
EDTA (disodium salt dihydrate, molecular biology grade, >99% purity)	MilliporeSigma/E5134-50G
Acrylamide (electrophoresis grade, ≥99% purity)	MilliporeSigma/A8887-100G
Bis-acrylamide (electrophoresis grade, ≥99% purity)	VWR/97061-142
Ammonium persulfate (electrophoresis grade, ≥98% purity)	VWR/0486-25G
TEMED	Bio-Rad/161-0800
Bromophenol blue	MilliporeSigma/B0126-25G
Xylene cyanol FF (molecular biology grade)	MilliporeSigma/X4126-10G
Glycerol (molecular biology grade)	MilliporeSigma/G5516-100ML
Positively charged nylon membrane	MilliporeSigma/11417240001
Gel-loading tips	Fisher Scientific/02-707-181

Tris Base (White Crystals or Crystalline Powder/Molecular Biology) **Fisher Scientific Catalog** #BP152-500

Boric acid **Merck MilliporeSigma (Sigma-Aldrich) Catalog** #B6768

EDTA disodium salt **Merck MilliporeSigma (Sigma-Aldrich) Catalog #**E5134-50G

Acrylamide **Merck MilliporeSigma (Sigma-Aldrich) Catalog #**A8887-100G

Bis-Acrylamide **VWR International (Avantor) Catalog #**97061-142

Ammonium persulfate (APS) ACS **VWR International (Avantor) Catalog #**0486-25G

TEMED (Tetra Methylene Diamine) **Bio-Rad Laboratories Catalog #**161-0800

Bromophenol Blue **Merck MilliporeSigma (Sigma-Aldrich) Catalog #**B0126-25G

Xylene Cyanol FF **Merck MilliporeSigma (Sigma-Aldrich) Catalog #**X4126-10G

Glycerol **Merck MilliporeSigma (Sigma-Aldrich) Catalog #**G5516-100ML

Nylon Membranes, positively charged **Merck MilliporeSigma (Sigma-Aldrich) Catalog #**11417240001

Gel-Loading Tips, 1-200µL **Fisher Scientific Catalog #**02-707-181

Chemiluminescence Immunoassay and Signal Detection (Steps 21–37):

A	B
**Materials**	**Vendor/Catalogue Number**
Maleic acid (≥99% purity)	Fisher Scientific/AC125231000
Sodium chloride (molecular biology grade, ≥99.8% purity)	VWR/TS32730-0010
TWEEN® 20 (molecular biology grade)	MilliporeSigma/P9416-50ML
Tris-HCl (molecular biology grade, >99% purity)	MilliporeSigma/648317-100GM
Sodium hydroxide, pellets	Various
Invitrogen™ I-Block™ Protein-Based Blocking Reagent	Fisher Scientific/T2015
Anti-Digoxigenin-AP, Fab fragments from sheep	MilliporeSigma/11093274910
CSPD (25 mM concentrate)	MilliporeSigma/11655884001
CDP-Star™ Substrate (12.5 mM Concentrate)	ThermoFisher/T2304

Maleic Acid, 99% **Fisher Scientific Catalog #**AC125231000

Tween-20 **Merck MilliporeSigma (Sigma-Aldrich) Catalog #**P9416-50ML

Tris, Hydrochloride, Molecular Biology Grade **Merck MilliporeSigma (Sigma-Aldrich) Catalog #**648317-100GM

I-Block™ Protein-Based Blocking Reagent **Thermo Fisher Scientific Catalog #**T2015

Anti-Digoxigenin-AP, Fab fragments **Merck MilliporeSigma (Sigma-Aldrich) Catalog #**11093274910

CSPD ready-to-use **Merck MilliporeSigma (Sigma-Aldrich) Catalog #**11655884001

CDP-Star™ Substrate (12.5 mM Concentrate) **Thermo Fisher Scientific** **Catalog** #T2304

INVASION ASSAY RECIPES (STEPS 1–5 & 38–39)

0.2 M EDTA solution (pH 8.0)

- 7.44 g disodium EDTA dihydrate

Dissolve disodium EDTA dihydrate in ~50 mL of diH_2O. Adjust pH to 8.0 with solid NaOH, then adjust volume to 100 mL with diH_2O. Filter (0.22 µm) or autoclave to sterilize and store at RT (<1 year, in a plastic bottle).

Note

Adapted from Ref. 1. At high concentrations, EDTA is poorly soluble in water and will only begin to dissolve between pH 7 and 8 ***[1]****. Slowly add NaOH before EDTA is fully dissolved. Heating the solution at ~60 °C and vigorously mixing with a stir bar can further facilitate dissolution. We recommend preparing EDTA solutions at least one day before intended use.*

2x Invasion Buffer (100 mM HEPES, 200 mM NaCl, 10 mM $MgCl_2$•6H_2O, pH 7.2, 20% sucrose, 2.88 mM spermine tetrahydrochloride)

- 238 mg HEPES
- 117 mg NaCl
- 20.3 mg MgCl2•6H_2O
- 2.00 g sucrose
- 10 mg spermine tetrahydrochloride

Dissolve HEPES, NaCl, and $MgCl_2$•6H_2O in ~5 mL of autoclaved diH_2O. Adjust pH to 7.2 with solid NaOH, then add sucrose and spermine tetrahydrochloride. Adjust volume to 10 mL with autoclaved diH_2O. Filter (0.22 µm) to sterilize, portion into 1.5 mL MCTs, and store at 2–4 °C (<1 year) or −10 to −20 °C (<5 years). Note that the final concentration will be halved in the Invasion Assay. Note that the final concentration will be halved in the Invasion Assay (50 mM HEPES, 100 mM NaCl, 5 mM MgCl2•6H_2O, pH 7.2, 10% sucrose, 1.44 mM spermine tetrahydrochloride).

Note

Filter before using after long-term storage.

EMSA AND BLOTTING RECIPES (STEPS 6–20)

0.5 M EDTA solution (pH 8.0)

- 93.05 g disodium EDTA dihydrate

Dissolve disodium EDTA dihydrate in ~300 mL of diH_2O. Adjust pH to 8.0 with solid NaOH, then adjust volume to 500 mL with diH_2O. Filter (0.22 µm) or autoclave to sterilize and store at RT (<1 year, in a plastic bottle).

Note

Adapted from Ref. 1. For additional comments in preparing EDTA solutions, see recipe notes for the 0.2 M EDTA solution above.

5x TBE Buffer (0.45 M Tris base, 0.45 M boric acid, 1 mM EDTA)

- 54.0 g Tris base
- 27.5 g boric acid
- 20 mL 0.5 M EDTA solution (pH 8.0)

Dissolve Tris base and boric acid in ~600 mL of diH_2O. Add 20 mL of 0.5 M EDTA solution (pH 8.0; see recipe above) and adjust volume to 1 L with diH_2O. Filter sterilize (0.22 µm); Store at RT (<1 year or until precipitation, see note).

Note

Adapted from Ref. 1. Concentrated TBE solutions will precipitate over time. If precipitation occurs, heat or microwave until precipitates dissolve or make fresh buffer. If convenient, immediately dilute to the working concentration (0.5x).

0.5x TBE Buffer (45 mM Tris base, 45 mM boric acid, 0.1 mM EDTA)

Dilute 100 mL of 5x TBE (recipe listed above) with 900 mL of diH_2O. Store at 2–4 °C (<6 months if autoclaved diH_2O is used).

Note

May also be stored at RT. However, the buffer should be cold before use in EMSAs. Prepare and chill at least 2 L (Mini-PROTEAN system, 4 gels and 1–2 blots) or 2.6 L (mPAGE gel system, 4 gels and 1–2 blots) before starting the protocol.

6x Loading Buffer (0.25% w/v bromophenol blue, 0.25% w/v xylene cyanol FF, 30% w/v glycerol)

- 12.5 mg bromophenol blue
- 12.5 mg xylene cyanol FF
- 1.5 mL glycerol

Combine bromophenol blue, xylene cyanol FF, and glycerol in a 15 mL centrifuge tube and vortex to dissolve. Adjust volume to 5 mL with autoclaved diH_2O. Portion into 1.5 mL MCTs and store at 2–4 °C (<1 year) or −10 to −20 °C (<5 years).

Note

Adapted from Ref. 1. Consult Table 2 for approximate migration of dyes in different concentrations of non-denaturing polyacrylamide gel.

A	B	C
**% Acrylamide**	**Bromophenol Blue**	**Xylene Cyanol FF**
8	45 nts	160 nts
12	20 nts	70 nts
15	15 nts	60 nts
20	12 nts	45 nts

Table 2. Approximate migration of bromophenol blue and xylene cyanol FF dyes in non-denaturing polyacrylamide gel
at different acrylamide concentrations. [a]

[a] Adapted from Ref. 2. "nts" = nucleotides.

40% Acrylamide/Bisacrylamide (19:1)

- 190 g acrylamide
- 10 g bis-acrylamide
- 500 mL autoclaved diH_2O

Carefully add acrylamide, bis-acrylamide, and autoclaved diH_2O to an amber-colored 500 mL bottle and swirl to dissolve. Store at 2–4 °C (<1 year).

Note

*Adapted from Ref. 1. Read section on SAFETY WARNINGS before handling acrylamide. Heat (37 °C) can be used to aid dissolution **[2]**. For long-term storage, especially at RT, filter sterilize (0.22 µm).*

APS Solution (10% w/v)

- 100 mg ammonium persulfate
- 1 mL autoclaved diH_2O

Add ammonium persulfate and autoclaved diH_2O to a 1.5 mL MCT. Vortex to dissolve.

Note

*Adapted from Ref. 2. APS solution is not stable at RT. If stored at 2–4 °C, it will remain stable for up to 12 h **[3]**, or even weeks **[2]**. We have found that the APS solution can be stored for at least 6 months with no noticeable change in catalytic activity when prepared as follows: make 10 mL of APS solution and portion into 0.5 mL and/or 1.5 mL MCTs. Fill each MCT almost to the top (limits air space while leaving room for expansion). Tightly seal the MCT lid, wrap with parafilm, place in a dark box, and store at −10 °C. Thaw to RT before use and store at 2–4 °C once opened. Dispose of APS solutions when gels take longer than 15 min to fully set.*

Polyacrylamide Gel Solution

A	B	C	D	E
	**8% (>90 nt)**	**12% (50–90 nt)**	**15% (25–50 nt)**	**20% (<25 nt)**
Autoclaved diH_2O	7 mL	6 mL	5.25 mL	4 mL
5x TBE [b]	1 mL	1 mL	1 mL	1 mL
40% Acrylamide/Bisacrylamide (19:1) [b]	2 mL	3 mL	3.75 mL	5 mL

Table 3. Recipes for gel solutions with different polyacrylamide concentrations. [a]

[a] Adapted from Ref. 1. [b] Recipe listed above.

Add all autoclaved diH_2O, 5x TBE, and 40% Acrylamide/Bisacrylamide to a 15 mL centrifuge tube and vortex to dissolve. Store at 2–4 °C (<6 months).

Note

Read section on SAFETY WARNINGS before handling acrylamide. The recipe makes enough solution for just over two gel casts (10.1 cm × 8.3 cm, 0.75 mm thickness). Any remaining gel solution in the tube (capped) can be used as an indicator for when gels are polymerized. Let refrigerated solutions warm to RT before casting as polymerization slows at cooler temperatures.

PAGE Gel Casts

When ready to cast, add 150 µL of APS Solution to the Polyacrylamide Gel Solution (recipes listed above). Briefly vortex (medium speed) to mix. Add 10 µL of TEMED. Briefly vortex (medium speed) to mix, then immediately transfer to gel casting plates using a P1000 or disposable transfer pipette. Once the gels are set, store at 2–4 °C (up to 1 month when stored properly, see note below).

Note

Decomposition of APS results in free radicals that initiate polymerization of acrylamide and bis-acrylamide. TEMED accelerates the formation of APS radicals, thereby catalyzing the polymerization reaction. Once APS and TEMED are added to the gel solution, work quickly to cast the gels. With fresh APS and TEMED solutions, gels will set within 15 min but full polymerization may take hours. Gels are considered to have set when (i) there is noticeable definition around the teeth of the gel comb and (ii) the remaining gel solution in the centrifuge tube has set (~30 min after casting). Once set, wrap individual gel plates in a paper towel, place in a Ziplock bag, soak with 0.5x TBE (recipe listed above), seal, and store at 2–4 °C (placed flat and not stacked). We recommend storing gels overnight before use to ensure complete polymerization.

CHEMILUMINESCENCE IMMUNOASSAY AND SIGNAL DETECTION RECIPES (STEPS 21–37):

The following recipes were adapted from Ref. 4. The volumes of the following buffers and solutions required for the immunological detection protocol will depend on the size of the membrane. Calculations for volumes are listed in the recipes and summarized in Table 4.

A	B	C	D	E	F
**1x Maleic Acid Buffer**	**1x Washing Buffer**	**Detection Buffer**	**1x Blocking Solution**	**Antibody Working Solution**	**Substrate Working Solution**
1 mL/cm^2 membrane	1 mL/cm^2 membrane	1 mL/cm^{2}membrane	1 mL/cm^2 membrane	1 µL per 10 mL Blocking Solution (75 mU/mL)	0.03 mL/cm^2 membrane.

Table 4. Buffer and solution volumes for the Chemiluminescence Immunoassay.

1x Maleic Acid Buffer (0.1 M maleic acid, 0.15 M NaCl, pH 7.5)

- 11.6 g maleic acid
- 8.70 g NaCl

Dissolve the maleic acid and NaCl in ~600 mL of diH_2O, then adjust pH to 7.5 with solid NaOH. Adjust the volume to 1 L with diH_2O. Autoclave to sterilize; store at RT (<6 months).

1x Washing Buffer (0.1 M maleic acid, 0.15 M NaCl, pH 7.5, 0.3% v/v TWEEN 20)

- 11.6 g maleic acid
- 8.70 g NaCl
- 3 mL TWEEN 20

Dissolve maleic acid and NaCl in ~600 mL of diH_2O, then adjust pH to 7.5 with solid NaOH. Adjust volume to 1 L with diH_2O. Autoclave to sterilize, then add TWEEN 20; store at RT (<6 months).

Detection Buffer (0.1 M Tris-HCl, 0.1 M NaCl, pH 9.5)

- 15.8 g Tris-HCl
- 5.84 g NaCl

Dissolve Tris-HCl and NaCl in ~600 mL of diH_2O then slowly adjust pH to 9.5 with solid NaOH. Adjust volume to 1 L with diH_2O. Filter sterilize (0.22 µm); store at RT (<6 months).

Note

Buffer must be used at the same temperature at which the pH was adjusted.

1x Blocking Solution

Calculate the required volume of 1x Blocking Solution (1 mL/cm^2 membrane). Calculate the amount of Invitrogen™ I-Block™ Protein-Based Blocking Reagent needed to obtain a 1% w/v solution in 1x Maleic Acid Buffer (recipe listed above). Dissolve the Blocking Reagent in 1x Maleic Acid Buffer while heating (<80 °C) and stirring vigorously with a magnetic stir bar (~45 min). Alternatively, heat the solution in a household microwave (~2 min, 700 W) in 10–30 sec intervals; use a stir rod to mix the solution between each interval.

Note

ALWAYS MAKE FRESH. Avoid heating up to a boil. Check volume after dissolution (buffer will evaporate partially while heating) and add more 1x Maleic Acid Buffer to reach required volume if needed. Cool to RT before use.

Antibody Working Solution

Calculate the required volume of Anti-Digoxigenin-AP Fab Fragment Solution needed for the antibody working solution (1 µL per 10 mL of 1x Blocking Solution). When prompted in the protocol (Step 24.4), add Anti-Digoxigenin-AP Fab Fragment Solution to the 1x Blocking Solution (recipe listed above) using a micropipette equipped with a low-retention pipette tip.

Note

ALWAYS MAKE FRESH. Centrifuge the Anti-Digoxigenin-AP Fab Fragment Solution at 10000 RPM for 5 min prior to addition. Use aseptic techniques when handling. Sterilize gloves, pipette, and working surface with 70% EtOH before use. Use autoclaved (low-retention) pipette tips when possible. We have noticed that the Anti-Digoxigenin-AP Fab Fragment Solution can adhere to the inside of the pipette tip. To ensure the Fragment Solution is fully expelled after addition to the 1x Blocking Solution, aspirate/dispense the solution a few times to flush the pipette tip.

Substrate Working Solution (0.25 mM of CSPD or CDP-Star in Detection Buffer)

Remove the substrate (CSPD or CDP-Star) from the refrigerator and let stand at RT for ~10 min (shielded from light) before opening. Calculate the required volume of Substrate Working Solution for the membrane size (0.03 mL/cm^2 membrane); calculate the volumes of substrate and Detection Buffer (recipe listed above) needed to achieve a 0.25 mM solution. In a 1.5 mL MCT (or other suitable size), add the calculated volume of substrate to the calculated volume of Detection Buffer, vortex to mix, and shield from light until ready to use.

Note

ALWAYS MAKE FRESH. Use aseptic techniques when handling. Sterilize gloves, micropipette, and working surface with 70% EtOH before use. Use autoclaved (low-retention) pipette tips when possible.

Safety warnings

SAFETY WARNINGS:

- Users should consult the safety/product data sheets, manuals, and other information provided by the manufacturers of the listed chemicals, reagents, and equipment prior to use.
- Users should wear suitable personal protective equipment (e.g., gloves, lab coat, eye protection) and have access to a well-ventilated workspace when conducting this protocol.
- Acrylamide is a potent neurotoxin (prior to polymerization) and should be handled carefully using appropriate personal protective equipment. When handling acrylamide, cover the working surface with a disposable absorbent pad/liner; change gloves often and use acrylamide-dedicated equipment (e.g., micropipettes and tip boxes) to reduce cross-contamination. Collect acrylamide-contaminated disposables in dedicated hazardous waste containers.

Before start

This protocol is sectioned into seven parts: (i) Invasion Assay, (ii) EMSA and Blotting, (iii) Chemiluminescence Immunoassay, (iv) Signal Detection and Quantification, (v) Dose-Response Invasion Assay, (vi) Data Visualization and C_{50} Calculations with R, and (vii) Suggestions for Increasing Throughput. Read the entire protocol prior to start. The entire protocol will take approximately 25–28 h, depending on the substrate used for immunological detection (Table 1). Suitable stopping points are indicated in the protocol.

A	B	C
**Part**	**Description**	**Time** [a]
Invasion Assay	DIG-labeling of DNA hairpin targets	25 min
	Preparation of ON probes for Invasion Assay	25 min [b]
	Invasion Assay	17 h
EMSA and Blotting	EMSA (i.e., resolution of recognition mixture)	4 h
	Blotting	45 min
Chemiluminescence Immunoassay	Wash 1	5 min
	Blocking of membrane	30 min
	Incubation with antibody	30 min
	Wash 2 and 3	30 min
	Dilution of substrate to working concentration	5 min
	Assembly of hybridization jacket	3 min
	Incubation with substrate (CDP-Star / CSPD)	10 min / 3 h 10 min
Signal Detection and Quantification	Capture of chemiluminescence signal	12 min
	Image processing and signal quantification	10 min
Data Visualization and C_{50} Calculations with R	Graphing dose-response data and calculating C_{50} values	10 min
	**Total time:**	**25 h / 28 h 10 min**

Table 1. List of steps and estimated time to complete the protocol.

Invasion Assay

17h 55m

1 Prepare 100 nM DIG-labeled DHP working solutions.

> **Note**
>
> Defrost all components and perform the entire procedure (except oven incubation in Step 1.2) On ice . Preheat oven to 37 °C before starting protocol.

1.1 In a 1.5 mL MCT, combine the components in the order listed in Table 2:

	A	B
	**Component**	**Amount**
	(1) 5x TdT reaction buffer	4 µL
	(2) 25 mM $CoCl_2$ solution	4 µL
	(3) DNA Hairpin to be labeled	100 pmol (variable volume)
	(4) 1 mM DIG-ddUTP	1 µL
	(5) Terminal transferase (400 U/µL)	1 µL
	(6) Autoclaved diH_2O	Volume to achieve 20 µL total solution

Table 2. Components and amounts for DIG-labeling assay.

1.2 Lightly vortex and briefly centrifuge the MCT to mix components. Incubate in oven at 37 °C for 00:15:00 , then place back On ice . Immediately add 2 µL of 0.2 M EDTA (pH 8.0; recipe listed under *Materials* tab) to stop the labeling reaction. Dilute to a 100 nM working concentration (i.e., dilution factor of ~45.5) with autoclaved diH_2O. Keep at -10 °C to -20 °C for long-term storage, or 2 °C – 4 °C when actively used to avoid repeated freeze-thaw cycles.

15m

Note

1. The Invasion Assay is performed in a total volume of 5 µL (i.e., 2.5 µL of a 100 nM DIG-labeled DHP solution + 2.5 µL of the single- or double-stranded ON probe in 2x Invasion Buffer). This quantity of DIG-labeled DHP (0.25 pmol) produces a sufficiently intense and reproducible signal for detection by the C-DiGit Blot Scanner, and is appropriate for the well size, gel thickness, and acrylamide percentage of the gel.
2. For instructive purposes, this protocol is written for an Invasion Assay involving a double-stranded probe (5'-ON/3'-ON) and the corresponding single-stranded probes (5'-ON only and 3'-ON only) at 25-fold molar probe excess (i.e., 0.25 pmol × 25 = 6.25 pmol probe) against a complementary DIG-labeled DHP target. Users should adjust probe concentrations as needed.

2 Preheat oven to 37 °C and heat block to 90 °C. Label five 0.5 mL MCTs (1–5). Add components to each MCT as listed in Table 3:

A	B	C
**MCT**	**Component**	**Purpose**
1	2.5 µL of 100 nM DIG-DHP	Lane 1 control
2	6.25 pmol of 5'-ON + 6.25 pmol of 3'-ON	Reaction vial, double-stranded probe
3	6.25 pmol of 5'-ON	Reaction vial, single-stranded probe
4	6.25 pmol of 3'-ON	Reaction vial, single-stranded probe
5	10 µL of 100 nM DIG-DHP	DIG-DHPs to be added to MCTs 2–4 (extra is prepared to account for potential pipetting errors)

Table 3. Components for Invasion Assay samples.

3 Completely dry down MCT 1–5 using a centrifugal vacuum concentrator, then resuspend as follows:

3.1 **MCT 1:** 2.5 µL of 2x Invasion Buffer (see recipe for “2x Invasion Buffer”) + 2.5 µL autoclaved diH_2O.

3.2 **MCT 2, 3, and 4:** 2.5 µL of 2x Invasion Buffer.

3.3 **MCT 5:** 10 µL of autoclaved diH_2O.

4 After resuspension, briefly centrifuge MCT 1–5 then anneal at 90 °C for 00:03:00 on a preheated heat block. Remove from heat and let cool to Room temperature (~ 00:05:00).

3m

5 Vortex and briefly centrifuge MCT 5. Add 2.5 µL of MCT 5 to MCT 2, 3, and 4. Briefly centrifuge then incubate MCT 1–4 for 17:00:00 at 37 °C .

17h

EMSA and Blotting

4h 45m

6 Approximately 45 min before the Invasion Assay is complete (i.e., ~16 h 15 min after placing samples in the oven), remove gel cast(s) from fridge and assemble the electrophoresis chamber (see recipe for "PAGE Gel Casts"; see illustration in Fig. 1 for chamber assembly). Pour cold 0.5x TBE Buffer (see recipe for "0.5x TBE Buffer") into the center of the electrode module(s) and check for leaks.

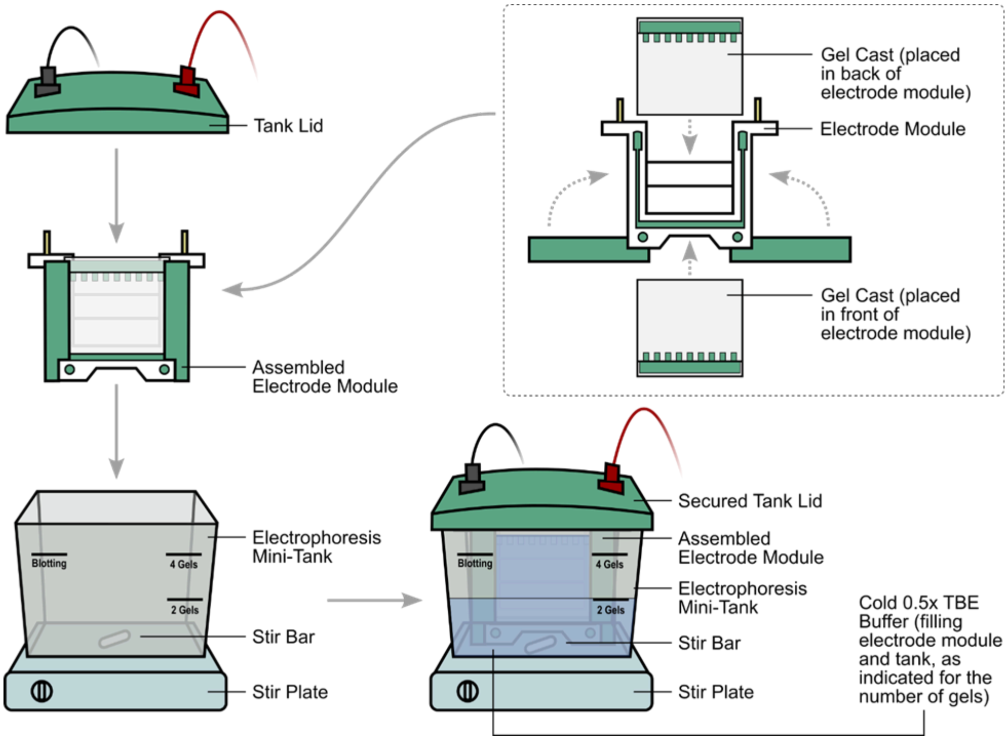

Figure 1. Illustration of assembling the electrophoresis chamber.

7 While submerged in 0.5x TBE Buffer, remove gel comb(s) from the gel cast(s) and use a transfer pipette or P1000 to carefully flush the wells with 0.5x TBE Buffer, ensuring each well is completely clear of residual gel and bubbles. Fill the chamber up to the indicated fill line with cold 0.5x TBE Buffer and equilibrate the gel(s) by running at 70 V and 4 °C for ~ 00:30:00 while vigorously stirring with a magnetic stir bar. 30m

8 When the Invasion Assay is complete, remove samples (MCT 1–4) from the oven and let sit ~ 00:15:00 . Briefly centrifuge samples to remove buffer from the lid and sides of the vials. 15m

9 Add 1 µL of 6x Loading Buffer (see recipe for “6x Loading Buffer”) to each MCT. Briefly centrifuge.

10 After gel equilibration, inspect the wells for bubbles or leftover gel residue, flush with 0.5x TBE Buffer if needed. Prepare to load the gel(s).

11 Use a P10 (equipped with a gel loading tip) to gently aspirate/dispense the sample in MCT 1 to mix, then load 5 µL into well 1 of the gel. Repeat for remaining samples, loading each into separate wells while taking care not to poke through the sides or bottoms of the wells with the gel loading tips.

12 Run loaded gel(s) at 70 V and 4 °C for 03:00:00 – 04:00:00 (or until the center of the two tracking dyes is 2/3 of the way down the gel plate) while vigorously stirring with a magnetic stir bar. Periodically check the mobility of the tracking dyes to ensure electrophoresis is stopped before samples run off the gel. 4h

13 Once complete, remove gel cast(s) from the electrophoresis chamber. Measure (i) the length of the gel and (ii) the distance from just above the top tracking dye to just below the bottom tracking dye (e.g., Fig. 2 illustrates a gel with width of 8.5 cm and tracking dye distance of 3.5 cm). Cut positively charged nylon membrane to match the measured dimensions.

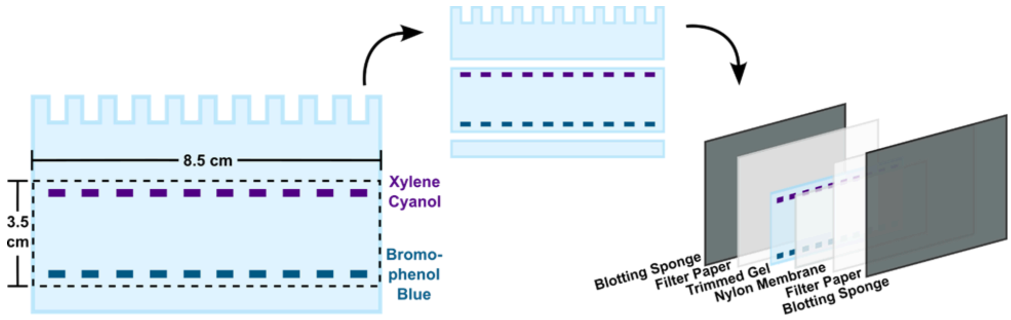

Figure 2. Assembly of blotting sandwich after EMSA. Dotted line highlights the bounds of the tracking dyes and gel edges for determining the minimum size of the nylon membrane and where to trim the gel before blotting. While the protocol thus far has described using only five lanes of the gel, use of all ten lanes is depicted in this illustration as more samples can be run simultaneously.

14 Trim four sheets of filter paper to the size of a blotting sponge. Stack two sheets of trimmed filter paper on one blotting sponge and soak with cold 0.5x TBE Buffer.

15 Using forceps, pick up the trimmed membrane (only touch the absolute edges) and place it on top of the 0.5x TBE Buffer-soaked filter paper/blotting sponge. Pour additional cold 0.5x TBE Buffer over the membrane/filter paper/blotting sponge to ensure the components are thoroughly soaked.

16 Hold the completed gel cast from Step 13 along the side edges and gently set the bottom edge of the gel cast against a soft surface (e.g., blotter pad). Angle the cast so that the short plate is slightly facing down. Using a gel separating tool, carefully pry apart the short and tall plates while guiding the gel off the tall plate. Once fully separated, the whole gel should be on the short plate; set the tall plate aside. If the gel proves difficult to transfer to the small plate and instead sticks to the tall plate, take note that the blot will be backwards after imaging (can be flipped in Image Studio software) and continue with the protocol.

17 Hover the gel-adhered plate over the 0.5x TBE Buffer-soaked membrane/filter paper/sponge with the gel directly facing the membrane (it will not fall off the glass plate). Align such that the tracking dyes in the gel are within the boundaries of the membrane. Gently place the gel on the membrane, taking care not to slide across the membrane or apply pressure. Carefully remove the gel from the plate using the gel separator tool and set aside the short plate. Use the gel separator tool to cut excess gel extending beyond the borders of the membrane (see Fig. 2 for example) and discard the cut-off pieces.

18 Place the other two sheets of trimmed filter paper (pre-soaked with cold 0.5x TBE Buffer) on top of the gel/membrane/filter paper/blotting sponge. Using a small glass test tube, gently roll over the top filter paper to remove air bubbles between the gel and membrane (see Fig. 3b for potential outcome if bubbles are not removed prior to blotting). Place a blotting pad (pre-soaked with cold 0.5x TBE Buffer) on top. The blotting sandwich is now fully constructed.

19 Secure the blotting sandwich in the gel holder cassette with the membrane side of the sandwich facing the clear side of the cassette and the gel side of the sandwich facing the black side of the cassette. Secure the cassette in a blotting electrode assembly, with the black side of the cassette facing the black side of the blotting electrode assembly. Assemble the blotting chamber with an ice pack and magnetic stir rod. Pour cold 0.5x TBE Buffer up to the indicated fill line on the electrophoresis mini tank, then run the blot for exactly 00:30:00 at 100 V and 4 °C , while vigorously stirring. 30m

Note

If the voltage reads less than 100 V after 30 min, increase the blotting time by 1 min per 2 V loss to avoid incomplete ON transfer (see Fig. 3b for incomplete transfer example).

20 Once the blot is complete, disassemble the blotting chamber and separate the blotting sandwich such that the membrane remains face-up (i.e., side with transferred ONs) on one of the filter paper/blotting sponges. Place the membrane/filter paper/blotting sponge in the UV Stratalinker and run exposure for 00:05:00 . The Invasion Assay products are now crosslinked to the membrane. 5m

Note

Good stopping point. Membranes can be placed and sealed in Ziploc bags then stored at 4 °C until ready for the Chemiluminescence Assay.

Chemiluminescence Immunoassay 1h 53m

21

Note

1. Unless otherwise specified, all incubations are performed at Room temperature with agitation (rotating or shaking).
2. Before starting the protocol, measure the dimensions of the membrane to determine the volume of each buffer and reagent needed (see Table 4 and "Additional Notes" under *Guidelines & Warnings tab* for more information).

A	B	C	D	E	F
**1x Maleic Acid Buffer**	**1x Washing Buffer**	**Detection Buffer**	**1x Blocking Solution**	**Antibody Working Solution**	**Substrate Working Solution**
1 mL/cm^2 membrane	1 mL/cm^2 membrane	1 mL/cm^2membrane	1 mL/cm^2 membrane	1 µL per 10 mL Blocking Solution (75 mU/mL)	0.03 mL/cm^2 membrane.

Table 4. Buffer and solution volumes for the Chemiluminescence Immunoassay.

22 Prepare the 1x Blocking Solution (see recipe for “1x Blocking Solution” and Table 4 for volume).

23 Set out three small containers (e.g., pipette boxes/lids) per membrane to be processed (see Fig. 3 for choosing an appropriately sized box). Label box #1 with “Wash”, box #2 with “Block/Ab.”, and box #3 with “Detection”.

24 After the 1x Blocking Solution has cooled to Room temperature , begin assay:

24.1 **Wash 1:** place membrane in “Wash” box and add 1x Washing Buffer (see recipe for “1x Washing Buffer” and Table 4 for volume). Incubate 00:05:00 . 5m

24.2 **Block:** transfer membrane to “Block/Ab.” box and add the 1x Blocking Solution prepared in Step 22. Incubate exactly 00:30:00 . Save Wash box and buffer for later. 30m

24.3 **Antibody prep:** ~ 00:06:00 before next step, centrifuge Anti-Digoxigenin-AP Fab Fragment Solution at 10000 rpm, 00:05:00 . 5m

24.4 **Antibody binding:** carefully pipette antibody (see recipe for "Antibody Working Solution" and Table 4 for volume) from the surface of the Anti-Digoxigenin-AP Fab Fragment Solution vial. Add directly to "Block/Ab." box. Incubate exactly 00:30:00 .

30m

24.5 **Wash 2:** transfer membrane back to Wash box (still containing 1x Washing Buffer used in Step 24.1). Incubate 00:15:00 .

15m

24.6 **Wash 3:** discard the used 1x Washing Buffer. Add fresh 1x Washing Buffer to the Wash box using the same volume as in Step 24.1. Incubate 00:15:00 .

15m

24.7 **Substrate prep:** clean working surface with 70% EtOH and ensure it is completely free of debris. Prepare Substrate Working Solution (see recipe for "Substrate Working Solution" and Table 4 for volume).

24.8 **Detection:** transfer membrane to "Detection" box and add Detection Buffer (see recipe for "Detection Buffer" and Table 4 for volume). Incubate 00:05:00 .

5m

Note

Membranes can remain in Detection Buffer (while rotating or shaking) for 01:00:00 .

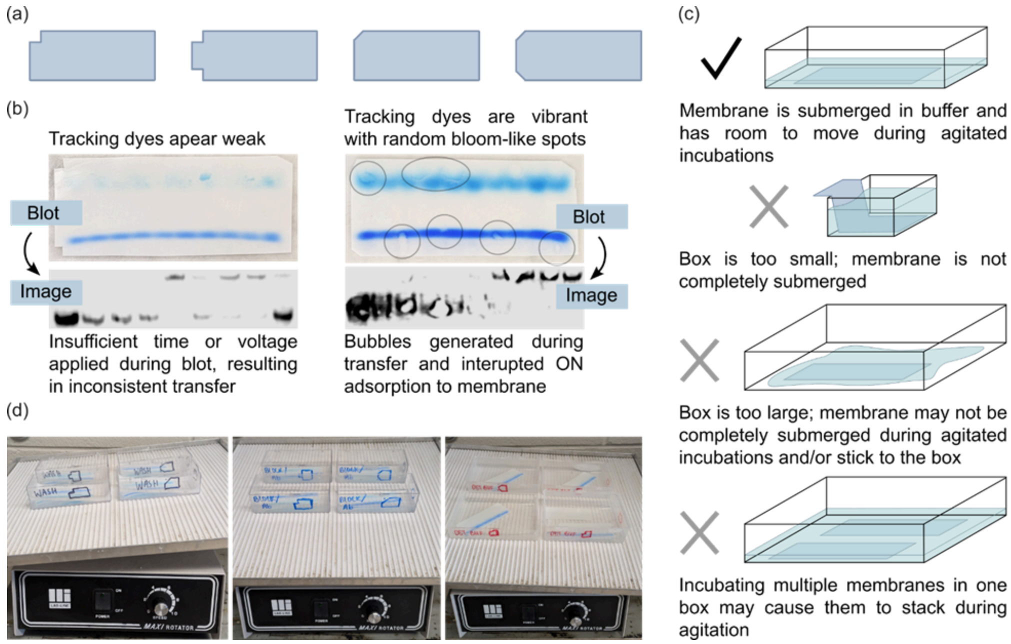

Figure 3. Illustrations and images pertaining to membranes during Blot and Chemiluminescence Immunoassay steps. (a) Suggested snip patterns to identify different blots when handling multiple membranes (discussed in Step 45.3 of protocol). (b) Images of failed blots that show how the appearance of the tracking dyes after blotting can indicate the efficiency of ON transfer. (c) Illustration of correct and incorrect boxes for immunoassay incubations. (d) Image of membrane incubations while rotating on the highest speed setting. Boxes are labeled with the membrane snip patterns (discussed in Step 46 of protocol) and incubation conditions (i.e., “Wash”, “Block/Ab”, and “DET Buf” for the Washing, Blocking and then Antibody Binding in the same box, and Detection steps of the protocol).

24.9 **Hybridization Jacket:**

1. Cut plastic wrap to be at least 2x larger than the membrane and lay flat on the clean working surface. Using forceps, remove membrane from “Detection” box and place it on the lower-half of the plastic wrap, facing up.
2. Use a P1000 to transfer the Substrate Working Solution (prepared in Step 24.7) to the membrane. Hover the pipette over the membrane and quickly expel the solution while continuously moving the pipette across the membrane (Fig. 4a).

Note

Failure to quickly expel the Substrate Working Solution across the membrane may result in background spots (see Fig. 4b for example).

3. Gently tilt membrane in every direction by carefully lifting individual edges of the plastic wrap (Fig. 4a). Ensure the Substrate Working Solution covers the entire surface. Take care to keep the solution on the membrane while tilting.
4. Hold the upper corners of the plastic wrap and bring them to a 90° angle with the top edge of the membrane. Tension should be taut, and the membrane should remain flat. Slowly move the plastic wrap towards you, allowing it to touch the top edge of the membrane. Continue to guide the plastic wrap down, slowly covering the membrane from the top edge to the bottom edge until it is completely covered (Fig. 4a).
5. Carefully use fingers or forceps to remove any bubbles or debris on top of the membrane by gently swiping across the plastic, taking care not to apply pressure that may damage the membrane.

Note

Bubbles on the surface of the membrane may interfere with the chemiluminescence reaction. If there are large or many bubbles, lift the plastic wrap off the membrane and repeat Step 24.9.4. Alternatively, use forceps or a blade to puncture a small hole in the plastic ~1 cm away from an edge of the membrane. Carefully guide the bubbles toward the hole to allow the air to escape. Take care to limit loss of substrate solution while doing so, and ensure the hole is covered during sealing.

6. Fold over each edge of the plastic wrap at least twice to seal the hybridization jacket and prevent the membrane from drying out during incubation (Fig. 4a). Fold over excess plastic wrap to about the size of the membrane, ensuring the solution drains towards the membrane as you fold.

24.10 **Initiate Reaction:** once assembled, place the hybridization jacket on a tray and cover with foil to shield from light. Incubate at 37 °C for 00:10:00 to initiate the reaction, then keep at Room temperature (away from light) for 03:00:00 (for CSPD substrate). If the CDP-Star substrate is used, the membrane can be imaged immediately following the 00:10:00 incubation at 37 °C .

3h 20m

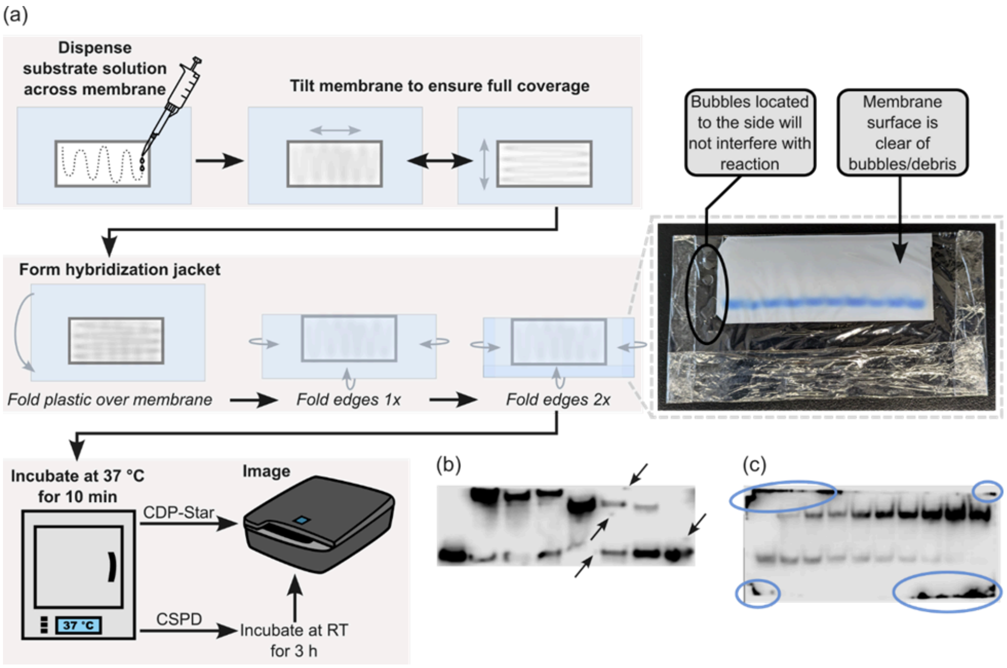

Figure 4. Illustration of (a) constructing and incubating the hybridization jacket; representative images of blots with high background signals (indicated by arrows or circles) from (b) applying Substrate Working Solution too slowly and (c) not wiping off excess CDP-Star Substrate Working Solution from membrane before imaging (Step 27, Note 1).

Signal Detection and Quantification

22m

25

Note

Before starting this protocol, follow the manufacturer's instructions for setting up the C-DiGit Blot Scanner and Licor Image Studio Software.

Approximately 10 min before Room temperature incubation is complete (when using the CSPD substrate) or right after placing the hybridization jacket in the oven (when using the CDP-Star substrate), open Image Studio on the computer and turn scanner on.

26 Gently clean the imaging surface of the scanner using a lint-free cloth and 70% EtOH, followed by autoclaved diH_2O. Ensure the glass surface is dry and free of debris.

27 Unwrap the hybridization jacket. Using **plastic** forceps, remove the membrane from the plastic wrap and place face-down onto the imaging surface.

Note

1. We have observed high background signals when the membrane is saturated with CDP-Star substrate solution during imaging (see Fig. 4c for example). To prevent this, gently wipe the membrane across the plastic wrap or lightly touch the corners with a lint-free cloth to remove excess substrate solution before setting it on the scanner. This can be ignored if CSPD substrate is used.
2. The imaging surface of the scanner is very delicate. Take extra care not to touch glass with plastic forceps nor to apply too much pressure when cleaning. Do not use metal forceps or anything that would easily scratch the scanner.

28 Close the scanner lid. In Image Studio, navigate to **Intensity** and select **High** > **Start.**

29 When imaging is complete, the software will prompt a selection of images that have been adjusted via default settings (one set of images adjusted in relation to contrast and then another set of images adjusted in relation to brightness). Choose an image in which all the bands seem visible. Manual adjustments for contrast and brightness will be done in Step 31.

30 Using plastic forceps, remove the membrane from the scanner. Clean the glass surface with autoclaved diH_2O and a lint-free wipe following imaging of each membrane. When all membranes have been imaged, clean the scanner with 70% EtOH and toss membranes.

Note

Good stopping point. Images can be saved and processed later.

31 To adjust the contrast and brightness of the image manually in Image Studio, navigate to and click the **Display** tab (on the right-hand side of screen) and select **Curves**. A single curve with three points overlaying a histogram will appear. Click and drag each of the points until background signals are reduced as much as possible while the faintest band is still visible. The leftmost point (slides left and right) will adjust the appearance of the background, the middle point (slides up and down) will adjust sharpness/softness, and the rightmost point (slides left and right) will adjust signal intensity across the entire image.

32 The leftmost band in the image should correspond to the DIG-DHP Lane 1 Control. However, if the blotting was performed backwards during Step 16, the DIG-DHP Lane 1 Control will appear as the rightmost band. If the blot is indeed backwards, navigate to and click the **Image** tab (top of screen). Navigate to the **Create** group and click **Rotate or Flip**. A selection of images that have been rotated and/or flipped will appear. Choose the image that places the DIG-DHP Lane 1 Control as the leftmost band.

33 Consider the positions of all bands in the image relative to the position of the DIG-DHP Lane 1 Control. Bands with the same mobility as the band in Lane 1 Control are determined to be unbound DIG-DHP target, while bands positioned above are recognition complexes.

34 Navigate to the **Analysis** tab (top of screen) and select **Add Rectangle**. Navigate to and click on the center of the leftmost band in the image, corresponding to the DIG-DHP Lane 1 Control. A rectangle outlining the band will automatically be applied and a number, corresponding to the signal of the band, will appear below the box. Repeat for all bands in the image.

Note

If there is a high amount of background in the image, the automatic "Add Rectangle" function may not accurately select the band and so the rectangle size will need to be manually adjusted. In the **Shapes** tab, click **Select** (to leave "Add Rectangle" function) and click on the rectangle you need to adjust. Click and drag on the corners or sides of the rectangle to adjust its position until the entire band is centered within the shape.

35 To subtract the background from signal quantification, remain in the "Analysis" tab and navigate to and select the leftmost icon in **Background**. From the drop-down menu, select **Average**, then change **Border Width** to "**1**", **Segment** to "**All**", and click **Save**. These settings will apply a thin border around each rectangle from Step 34. The pixels within that border will be quantified as a background value that is then subtracted from the band's signal. As such, the values under each rectangle will update to the background-corrected values after completing this step.

36 Record the background-corrected signal values for each band and calculate the percent recognition by each probe: ((signal of recognition complex) / (signal of unbound target + signal of recognition complex)) × 100.

37 Repeat above sections of the protocol until a minimum of three independent experiments have been performed. Calculate the average percent recognition and standard deviation

for each probe.

Calculate the sample standard deviation using the Excel *STDEV* function or the following formula:

$$s = \sqrt{\frac{\sum(x-\bar{x})^2}{(n-1)}}$$

where s is the sample standard deviation, x is an individual data point, $\bar{x}$ is the sample mean, and n is the total number of data points in the sample.

Dose-Response DIG-DHP Invasion Assay

38

Note

Once an initial fixed-dose Invasion Assay has been performed (e.g., 25-fold molar probe excess, as described in Steps 2–5), a study might call for evaluation of a subset of dsDNA-targeting probes using dose-response assays to determine C_{50} values (i.e., the probe concentration resulting in 50% recognition of DHP targets). If so, evaluate the results from the fixed-dose Invasion Assay to determine a proper concentration gradient for the probes. For a 10-well gel, nine different concentrations of ON probes would be used for the dose-response assay; well 1 is dedicated to the control DIG-DHP (see the dose-response data presented in Fig. 4 of the associated manuscript for example doses). Ideally, the highest probe concentration used in the dose-response assay will result in complete recognition of the DIG-DHP target, while the lowest probe concentration will result in minimal or no recognition. However, some probes may exhibit very low affinity to the target, thereby requiring extremely high concentrations for full recognition (e.g., 200-fold molar probe excess). If this is the case and access to the probe is limited, choose a reasonable concentration to be the highest concentration sample in the assay.

39 Follow the entire protocol as instructed but adjust probe volumes needed for selected probe quantities (i.e., perform the Invasion Assay (Steps 2–5) with variable concentrations of a probe). Calculate the average percent recognition and standard deviation for each concentration from at least three independent experiments, as described in Step 36 and 37.

Data Visualization and C_{50} Calculations with R

10m

40

> **Note**
>
> Companion code can be accessed at https://github.com/MeEverly/DNA-Invasion-Protocol. Review "README.md" and lines 1–33 of "DR_Script.R" for information on the packages and algorithms used for calculating C_{50} values and displaying dose-response graphs.

Set up a CSV file exactly as follows:

40.1 **A1:** use for titles, notes, or any identifying information you want in the file (entire row is skipped in code). Leave blank otherwise.

40.2 **A2–D2:** label exactly as shown in Fig. 5.

40.3 **A3–A11:** concentrations used for dose-response assays, listed in ascending order.

40.4 **B3–B11:** the average percent recognition observed for each concentration listed in column A (see Step 36 for calculation).

40.5 **C3–C11:** the standard deviation for each response listed in column B (see Step 37 for calculation).

40.6 **D2–D11:** the probe name responsible for the data in columns B and C.

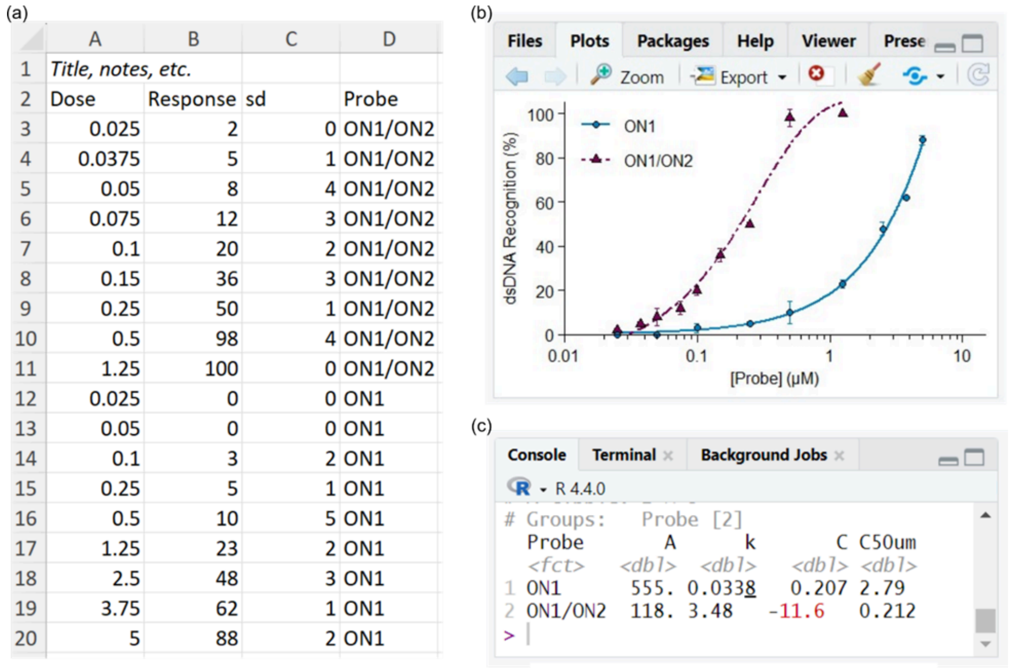

	A	B	C	D
1	Title, notes, etc.			
2	Dose	Response	sd	Probe
3	0.025	2	0	ON1/ON2
4	0.0375	5	1	ON1/ON2
5	0.05	8	4	ON1/ON2
6	0.075	12	3	ON1/ON2
7	0.1	20	2	ON1/ON2
8	0.15	36	3	ON1/ON2
9	0.25	50	1	ON1/ON2
10	0.5	98	4	ON1/ON2
11	1.25	100	0	ON1/ON2
12	0.025	0	0	ON1
13	0.05	0	0	ON1
14	0.1	3	2	ON1
15	0.25	5	1	ON1
16	0.5	10	5	ON1
17	1.25	23	2	ON1
18	2.5	48	3	ON1
19	3.75	62	1	ON1
20	5	88	2	ON1

Figure 5. Screenshots of example dose-response data and R script output. (a) CSV file set-up. (b) Plot generated and (c) C_{50} output in RStudio Console upon execution of the Dose-Response R script ("DR_Script.R") using the data shown in (a). A, k, and C are fitting constants generated by the algorithm. Data was fabricated for instructive purposes.

41 To include additional dose-response profiles on the same graph, insert the respective data directly below the first set and use a unique name for the Probe column (e.g., see column D and rows 12–20 in Fig. 5a).

42 Open the "DR_Script.R" file from the GitHub repository and download or copy the raw code into RStudio. Follow the instructions listed in lines 20–26. Upon execution, the calculated C_{50} values will be printed in the RStudio Console (e.g., Fig. 5c).

Suggestions for Increasing Throughput

43

> **Note**
>
> The electrophoresis mini tanks used in this protocol hold up to four gels. Thus, it is possible to run multiple gels simultaneously. The following notes are recommendations on how to effectively organize, conduct, and process multiple assays during each step of the protocol.

General Suggestions for Organizing Assays:

43.1 Dedicate a number code to individual Invasion Assays and a letter code to replicates (e.g., GEL001a, GEL001b, GEL001c).

43.2 Color-code assay replicates to save time with labeling vials and aid in locating/organizing samples during assay prep.

43.3 Before starting any assays, print and fill out the provided Gel ID sheets (see File S4 of associated manuscript or file attachment).

44 **DIG-DHP Invasion Assay:**

44.1 As noted previously, percent recognition is reported as an average from at least three independent replicates (i.e., three separate, but identical, trials). Sample preparation of all three replicates (Steps 2–3.3, prior to annealing and combining probes with target) can be done in advance, then stored at 2 °C – 4 °C until needed. Meticulously label the MCTs to keep samples organized (see Fig. 6 for example). When you are ready to continue the protocol for specific replicate(s) after storage, briefly centrifuge samples to remove buffer from the lid and sides of the vials and continue to Step 4 of the protocol.

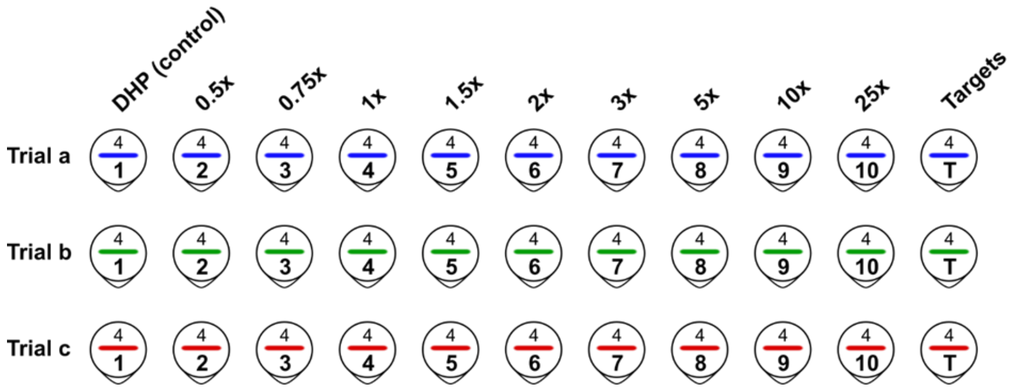

Figure 6. Example MCT set-up for three replicates of a Dose-Response Invasion Assay. Color coding (blue, green, or red middle line) is used to easily identify and organize samples. Numbers above the line correspond to the Gel ID Sheet (e.g., Gel ID "4") and numbers below the line correspond to the gel lane number in which the samples will be added to. Samples are added to MCTs in accordance with the Gel ID Sheet.

44.2 When annealing (Step 4), start a stopwatch the moment the first MCT is placed on the hotplate. Continue placing each MCT one-at-a-time at a consistent rate. When the stopwatch reads 3 min, remove each MCT in the order they were placed while matching the rate at which they were set in the hotplate.

45 **EMSA and Blotting:**

45.1 Complete Steps 6–12 with four gels (i.e., four sets of Invasion Assays). Write each Gel ID on the outside of the electrophoresis mini tank, near the respective gel, to identify the gels after electrophoresis. Load all four gels quickly to limit diffusion of samples up the wells.

45.2 After electrophoresis, prepare two blots at a time (i.e., prepare one blotting chamber). If blotting equipment is limited, leave the finished gels in their electrophoresis chambers (not connected to the power supply) until the first blotting is complete.

45.3 Snip the edges of membranes (where there is no possibility of ONs bound) in unique patterns to differentiate experiments (see Fig. 3a for example). Draw the snip patterns on the corresponding Gel ID Sheets.

46 **Chemiluminescence Immunoassay and Signal Detection:**

46.1 Operate protocol with up to eight blots at a time. When handling multiple blots, always handle the blots in the same order to reduce variations in incubation times. Number the order on the boxes if needed.

46.2 Draw membrane snip patterns on assay boxes to quickly identify and switch membranes between boxes during the immunoassay procedure.

46.3 Pre-aliquot buffers in 50 mL centrifuge tubes and pour into assay boxes when needed. If the buffer volumes are different, draw snip patterns on tubes for quick identification.

46.4 Multiple membranes may be imaged simultaneously if they fit within the imaging window of the C-DiGit Blot Scanner.

Note

When using CSPD, we suggest preparing hybridization jackets at the same time and then completing all images within the 3–4 h window of peak emission. CDP-Star, however, exhibits a much greater signal output such that images can be captured immediately following 00:10:00 incubation at 37 °C . We have noticed increased background signals, particularly around the edges of the membranes, with longer incubation times. Thus, when multiple membranes are to be imaged and CDP-Star is the substrate, we suggest preparing hybridization jackets only as needed to avoid extended reaction times.

Protocol references

1. Common Buffers and Stock Solutions. Current Protocols in Nucleic Acid Chemistry. 2000;00(1):A.2A.1-A.2A.12.
2. J. Sambrook. Molecular cloning: a laboratory manual. 2nd ed. Cold Spring Harbor, N.Y: Cold Spring Harbor Laboratory; 1989.
3. Sigma-Aldrich Inc. Ammonium Persulfate [Internet]. Saint Louis, Missouri; Available from: https://www.sigmaaldrich.com/deepweb/assets/sigmaaldrich/product/documents/277/489/a3678pis.pdf
4. Roche. DIG Luminescent Detection Kit [Internet]. Mannheim, Germany; Available from: https://www.sigmaaldrich.com/deepweb/assets/sigmaaldrich/product/documents/680/343/11363514910.pdf
5. Applied Biosystems. CSPD® and CDP-Star® substrates for alkaline phosphatase [Internet]. MA, USA; 2001. Version 9026.A.2. Available from: **https://assets.fishersci.com/TFSAssets/LSG/manuals/cms_041790.pdf**
