# Supplementary material for: An electrophoretic mobility shift assay with chemiluminescent readout to evaluate DNA-targeting oligonucleotide-based probes
Source: PLoS One. 2025 Oct 30;20(10):e0335674. doi: 10.1371/journal.pone.0335674 (PMC12574872; doi:10.1371/journal.pone.0335674)
Supplement: S2 File — (PDF) [file pone.0335674.s002.pdf]

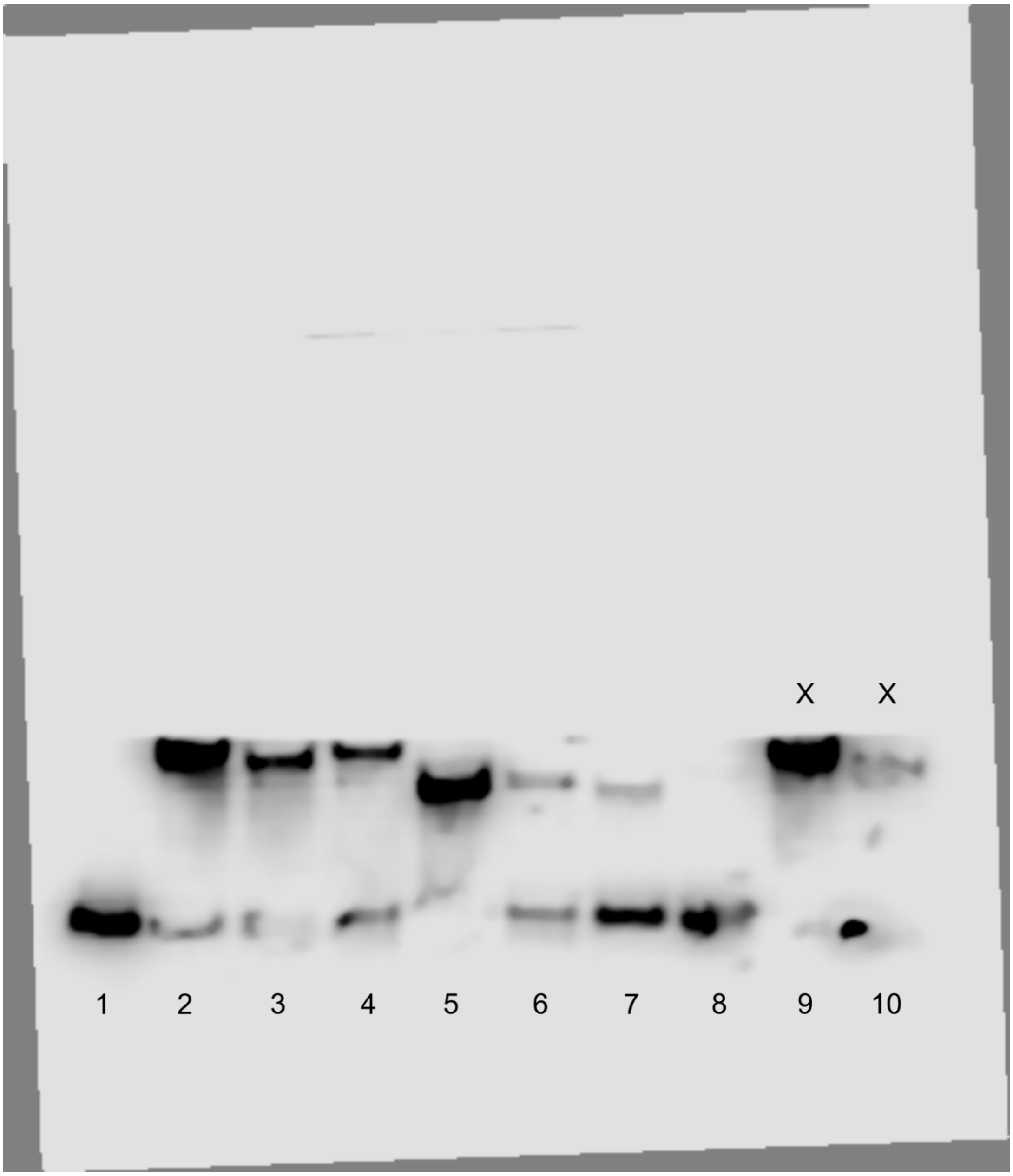

Fig 1. Annotated raw image of gel electrophoretogram shown in Fig 3b of the main manuscript. The full image was processed using Image Studio Digits Ver 5.2 software. The image was subjected to the "Noise Removal" operation (1x). Contrast was then adjusted such that background signals were reduced but all bands remained visible. Lanes are numbered according to loading order. "X" indicates lanes that are not included in the final figure. The faint horizontal line above the electrophoretogram is from the surface of the scanner and not the blot.

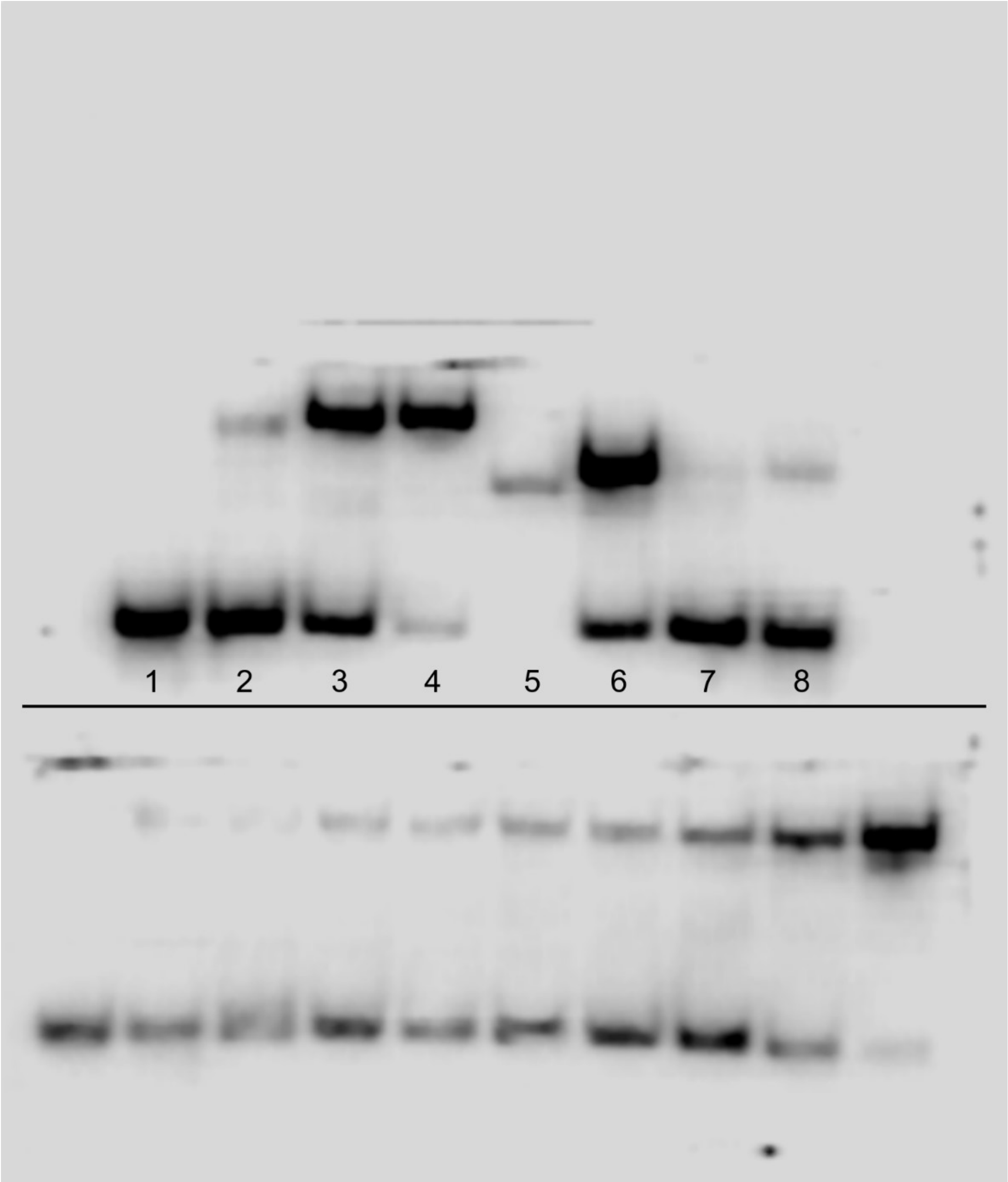

Fig 2. Annotated raw image of gel electrophoretogram shown in Fig 3c of the main manuscript. The full image was processed as described in Fig 1. Lanes are numbered according to loading order. The electrophoretogram below the solid black line is from a separate blot (image not used in this paper) that was simultaneously imaged with the respective blot. The faint horizontal line above the electrophoretogram is from the surface of the scanner and not the blot.

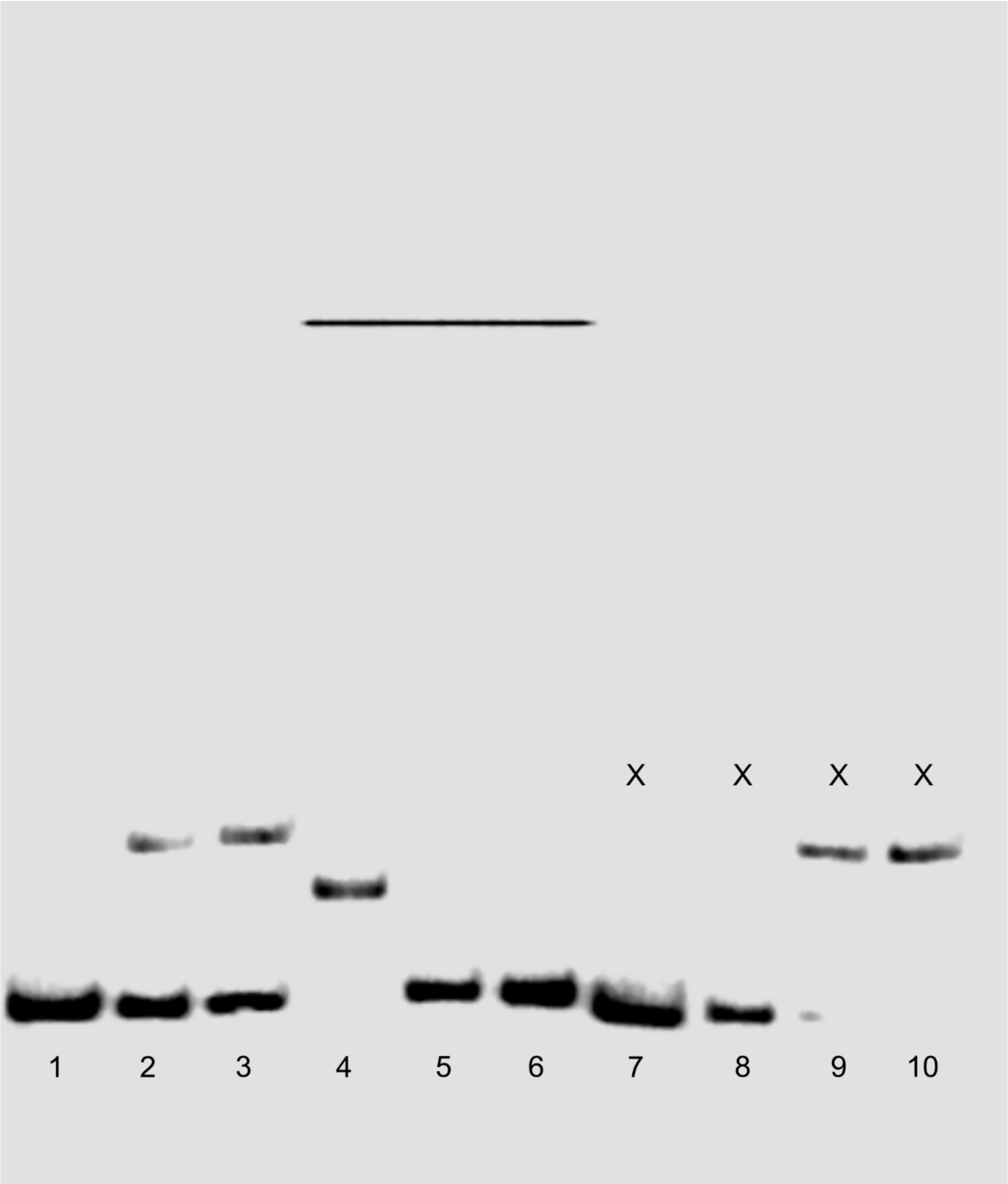

Fig 3. Annotated raw image of gel electrophoretogram shown in Fig 3d of the main manuscript. The full image was processed as described in Fig 1. Lanes are numbered according to loading order. “X” indicates lanes that are not included in the final figure. The horizontal line above the electrophoretogram is from the surface of the scanner and not the blot.

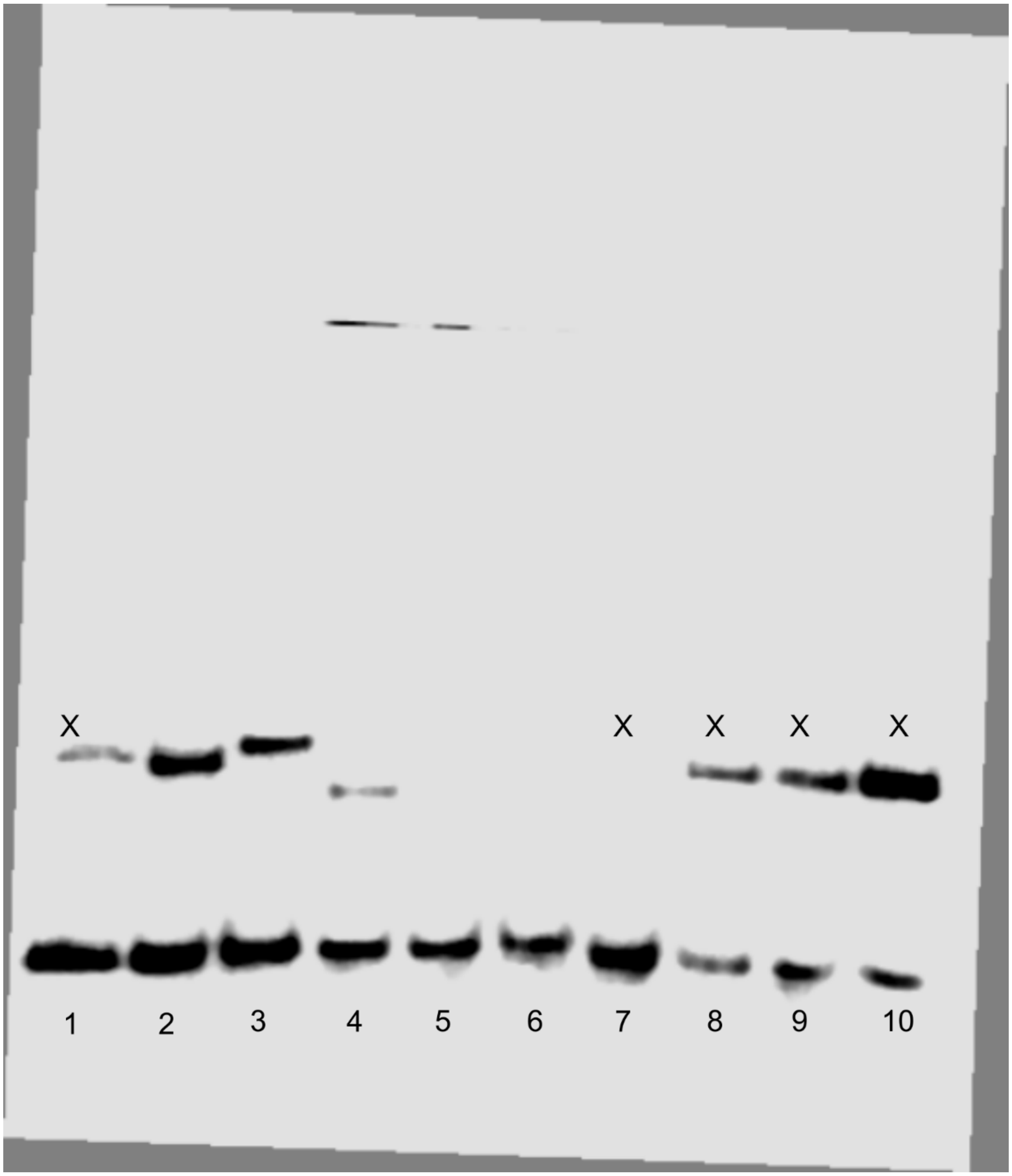

Fig 4. Annotated raw image of gel electrophoretogram used for the composite shown in Fig 3e of the main manuscript. The full image was processed as described in Fig 1. Lanes are numbered according to loading order. "X" indicates lanes that are not included in the final figure. The horizontal line above the electrophoretogram is from the surface of the scanner and not the blot.

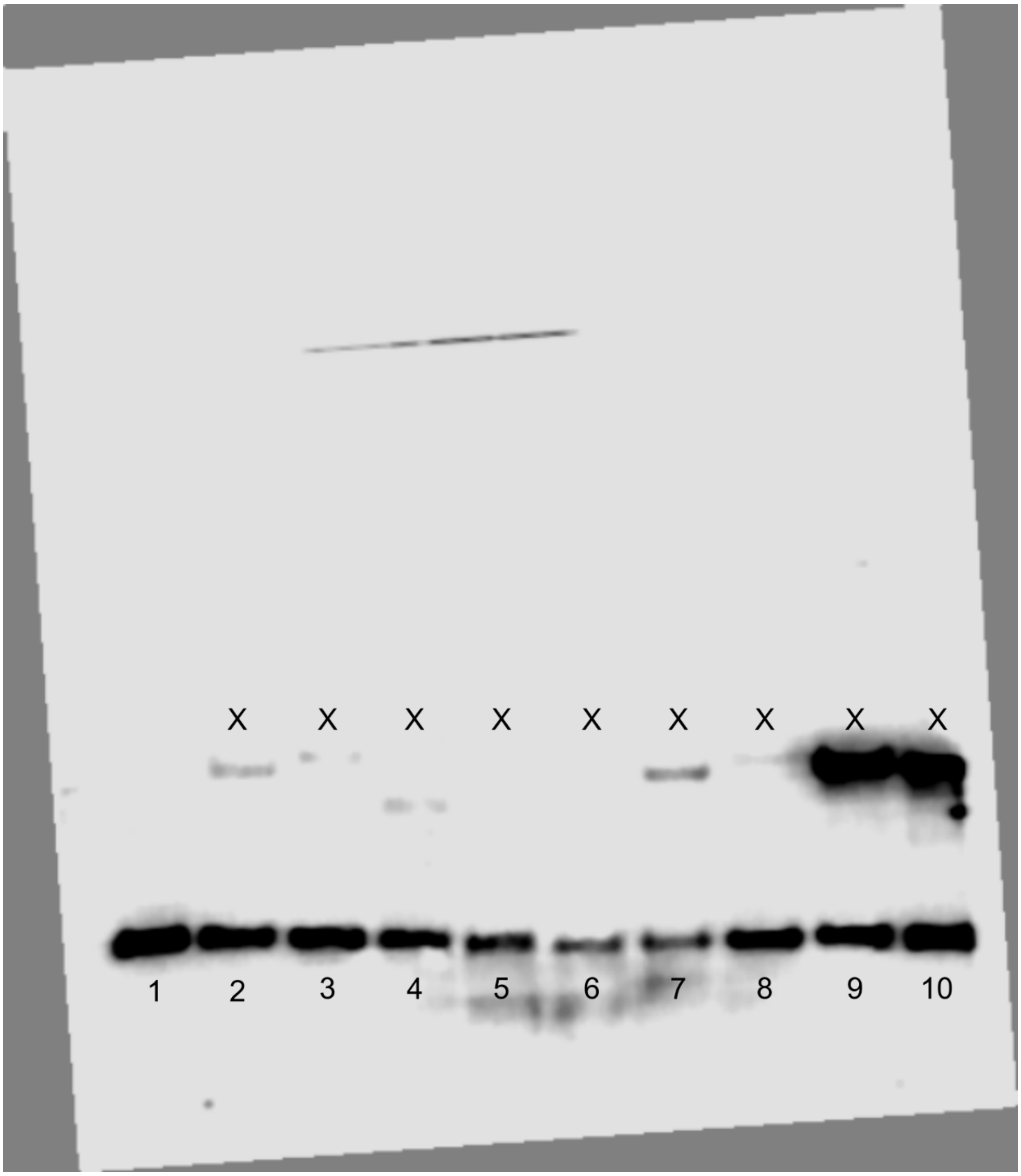

Fig 5. Annotated raw image of gel electrophoretogram used for the composite shown in Fig 3e of the main manuscript. The full image was processed as described in Fig 1. Lanes are numbered according to loading order. "X" indicates lanes that are not included in the final figure. The horizontal line above the electrophoretogram is from the surface of the scanner and not the blot.

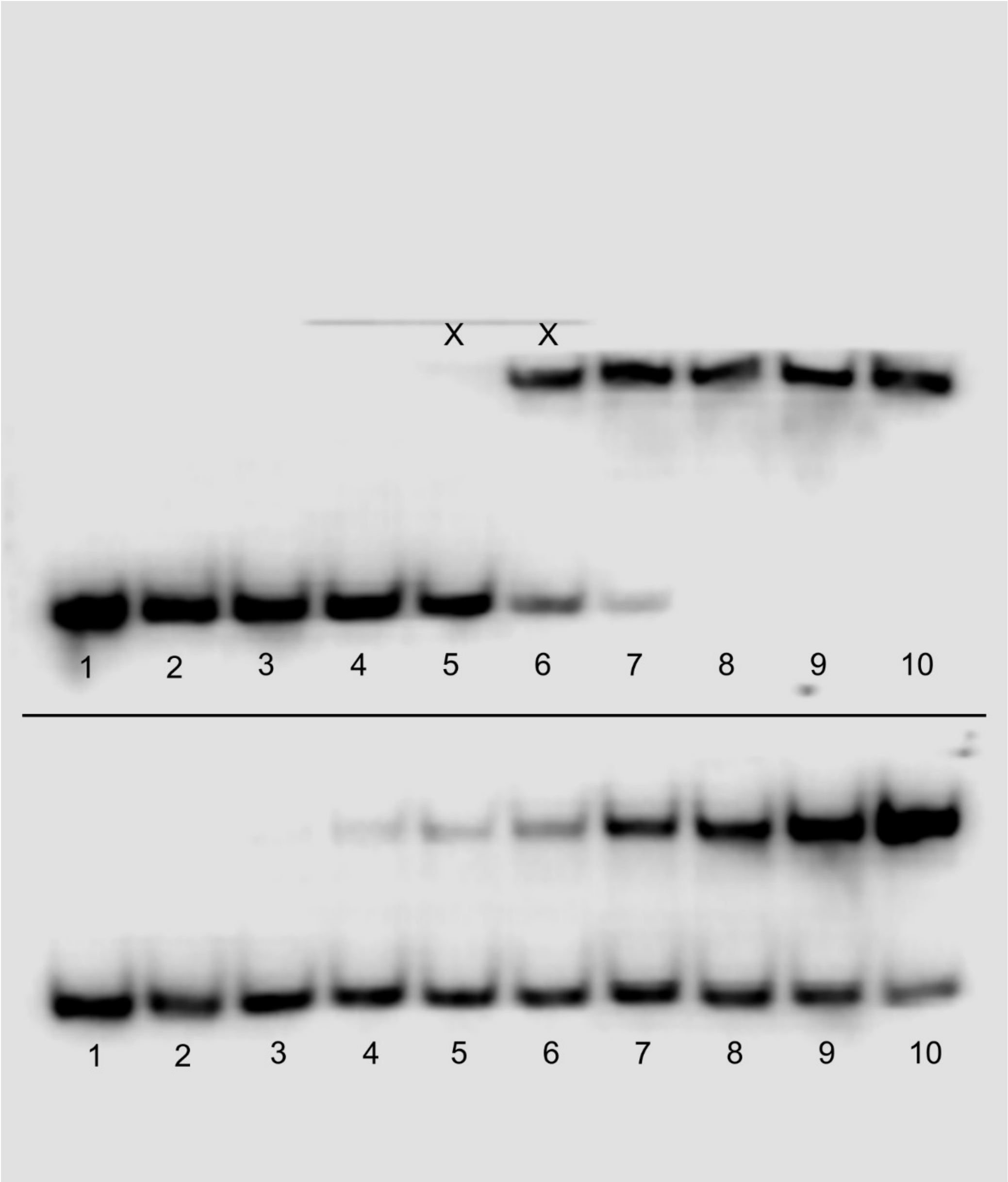

Fig 6. Annotated raw image of gel electrophoretogram shown in Fig 4b (bottom) and the composite shown in Fig 4c (top) of the main manuscript. The full image was processed as described in Fig 1. Lanes are numbered according to loading order. The horizontal line above the top electrophoretogram is from the surface of the scanner and not the blot.

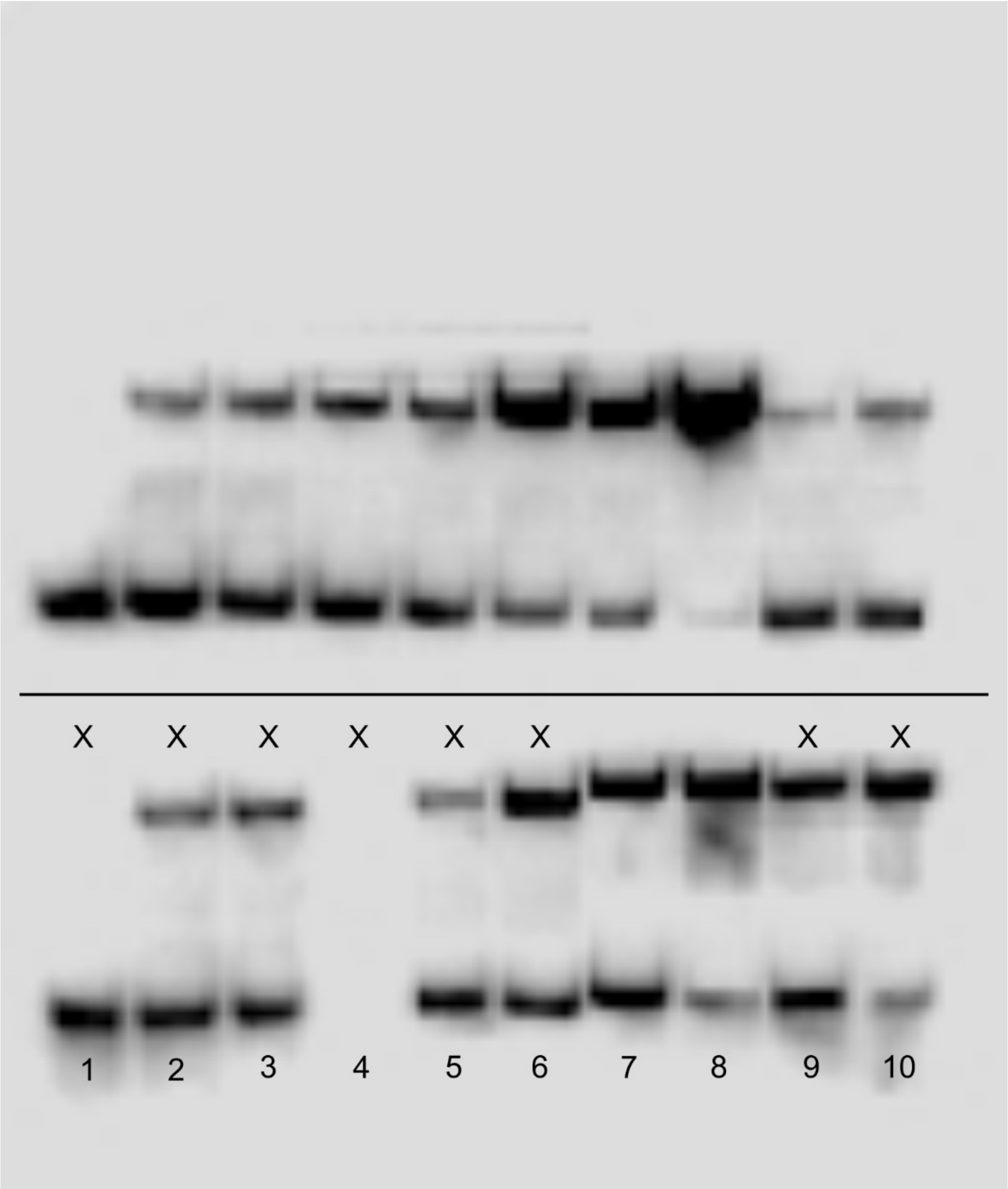

Fig 7. Annotated raw image of gel electrophoretogram used for composite shown in Fig 4c of the main manuscript. The full image was processed as described in Fig 1. Lanes are numbered according to loading order. The electrophoretogram above the solid black line is from a separate blot (image not used in this paper) that was simultaneously imaged with the respective blot. The faint horizontal line above the electrophoretogram is from the surface of the scanner and not the blot.

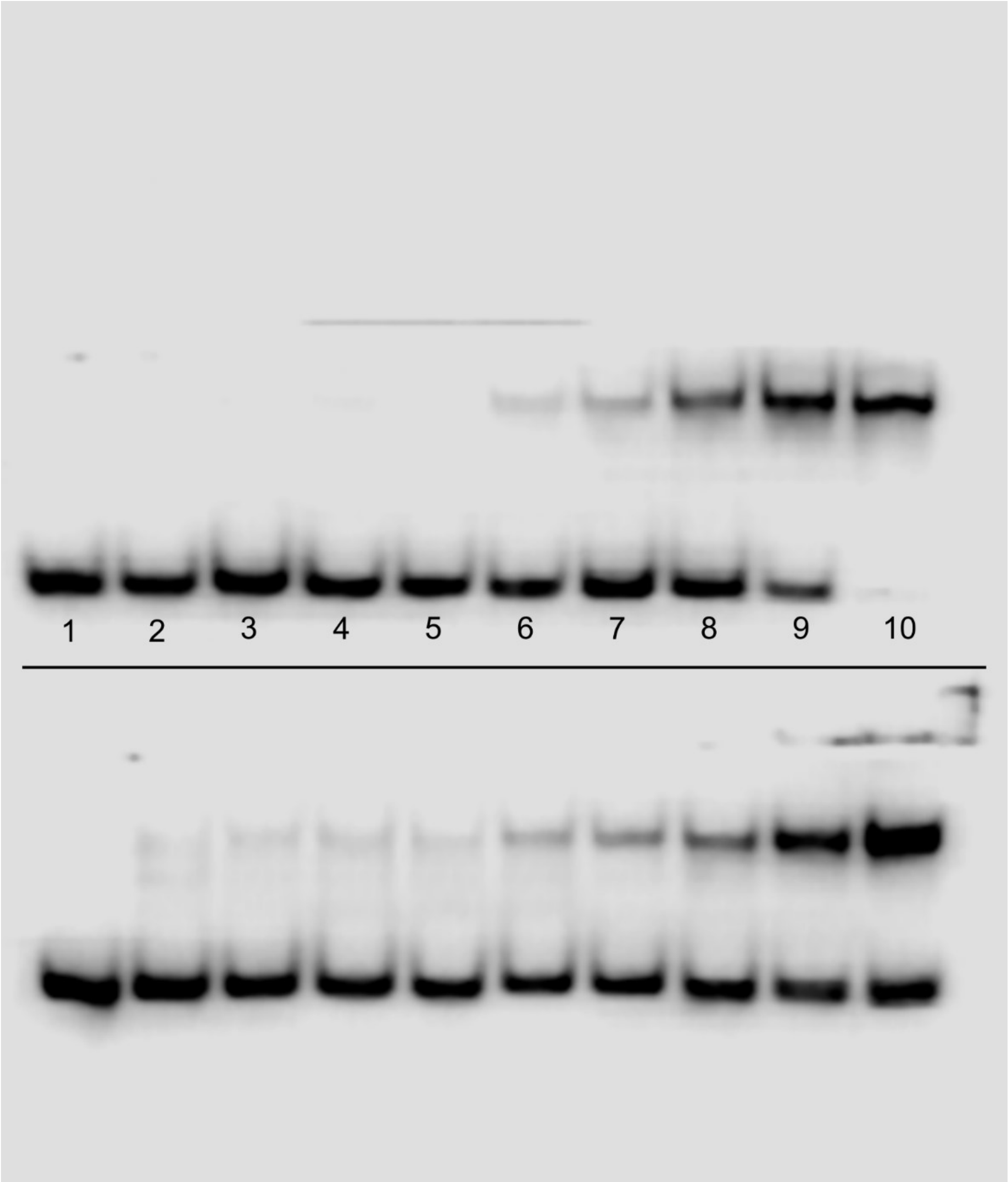

Fig 8. Annotated raw image of gel electrophoretogram shown in Fig 4d of the main manuscript. The full image was processed as described in Fig 1. Lanes are numbered according to loading order. The electrophoretogram below the solid black line is from a separate blot (image not used in this paper) that was simultaneously imaged with the respective blot. The horizontal line above the electrophoretogram is from the surface of the scanner and not the blot.

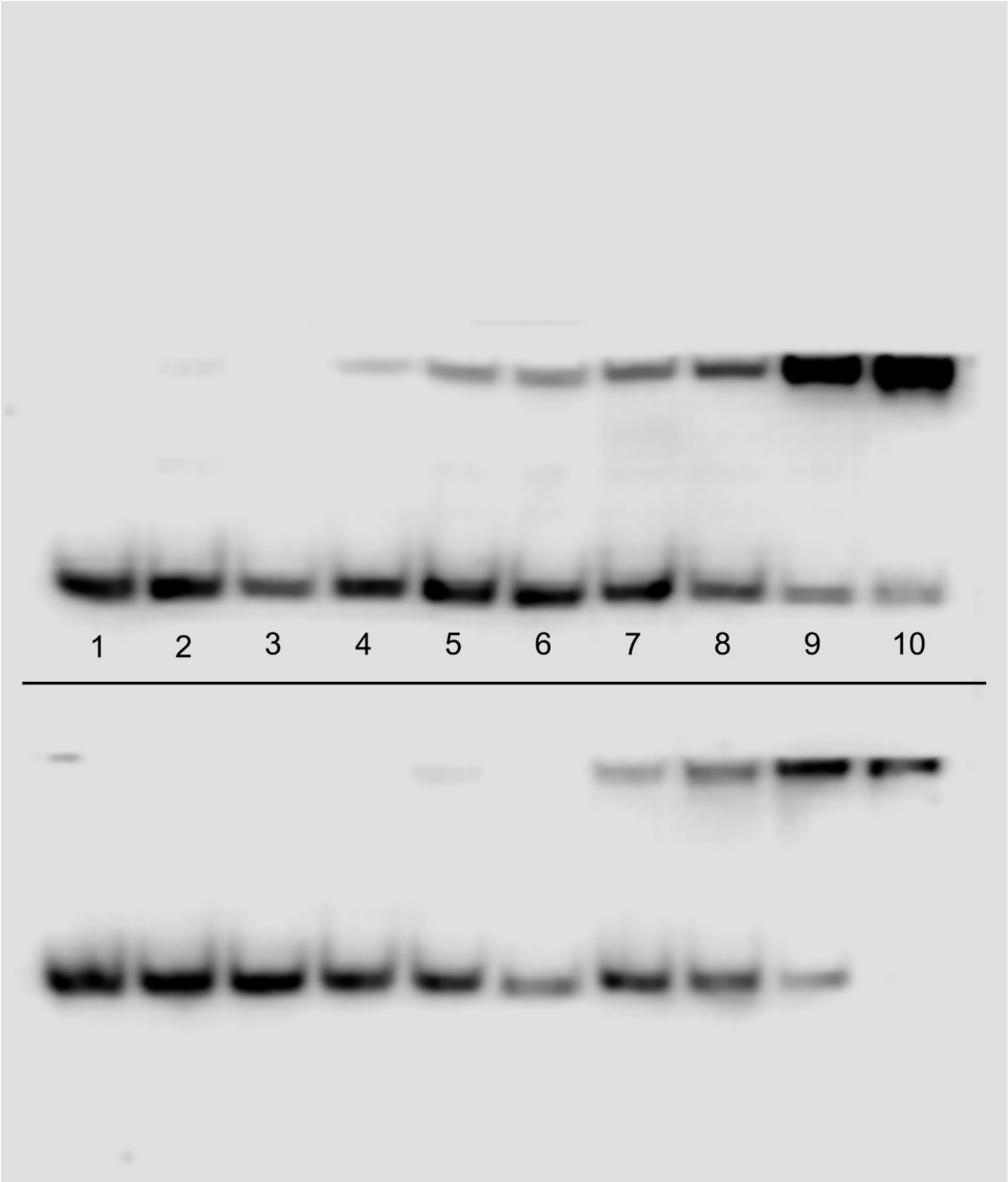

Fig 9. Annotated raw image of gel electrophoretogram shown in Fig 4e of the main manuscript. The full image was processed as described in Fig 1. Lanes are numbered according to loading order. The electrophoretogram below the solid black line is from a separate blot (image not used in this paper) that was simultaneously imaged with the respective blot. The faint horizontal line above the electrophoretogram is from the surface of the scanner and not the blot.
