# Supplementary material for: An electrophoretic mobility shift assay with chemiluminescent readout to evaluate DNA-targeting oligonucleotide-based probes
Source: PLoS One. 2025 Oct 30;20(10):e0335674. doi: 10.1371/journal.pone.0335674 (PMC12574872; doi:10.1371/journal.pone.0335674)
Supplement: S3 File — (DOCX) [file pone.0335674.s003.docx]

An electrophoretic mobility shift assay with chemiluminescent readout to evaluate DNA-targeting oligonucleotide-based probes.

Michaela E. Everly^1^, Peter J. Wieber^1^, Ibrahim Al Janabi^1^, Patrick J. Hrdlicka^1*^

^1^ Department of Chemistry, University of Idaho, Moscow, Idaho 83844-2343, USA

^*^ Corresponding author

E-mail: hrdlicka@uidaho.edu (PJH)

**S3 File: Supporting Data**

| ESI-MS spectra and HPLC chromatograms of LNA probes used herein (Table 1 and Figs 1‒6) | 3 |
| --- | --- |
| Method – synthesis, purification, and characterization of LNA probes | 10 |
| Method – thermal denaturation and UV-Vis absorption experiments | 11 |
| Representative thermal denaturation profiles and differential curves (Fig 7) | 12 |
| Representative thermal denaturation profiles for single-stranded LNA probes (Fig 8) | 13 |
| Sequences, *T*_m_ and TA values of chimeric LNA:LNA, conventional Invader, and LNA probes (Table 2) | 14 |
| Discussion of thermal denaturation results | 16 |
| Discussion and data regarding UV-Vis absorption experiments of probes containing Invader modifications (Table 3 and Fig 9) | 18 |
| Method – control experiment evaluating CDP-Star concentration-to-signal linearity and LOD for the C-DiGit Blot Scanner | 21 |
| Results for the control experiment evaluating CDP-Star concentration-to-signal linearity and LOD for the C-DiGit Blot Scanner (Fig 10) | 22 |
| Additional discussion on the dsDNA-targeting properties of probes evaluated herein | 22 |
| Supplementary references | 24 |

**Table 1.** **ESI-MS data of LNAs synthesized for the present study.** *^a^*

| **ON** | **Sequence** | **Calculated *m*/*z* (M-H)^-^** | **Observed *m*/*z* (M-H)^-^** |
| --- | --- | --- | --- |
| LNA1d | 5'-aacagttctatcaG | 4653 | 4653 |
| LNA1u | 5'-ctgatagaactgtT | 4670 | 4672 |
| LNA2d | 5'-atcaagagccatgC | 4663 | 4666 |
| LNA2u | 5'-gcatggctcttgaT | 4676 | 4678 |
| LNA3d | 5'-atcgccaataacgA | 4661 | 4664 |
| LNA4d | 5'-tttcaaatggcatA | 4654 | 4655 |

*^a^* LNA monomers are denoted in lower case letters (“c” = 5-methyl-cytosin-1-yl LNA monomer), DNA monomers are denoted in upper case letters.


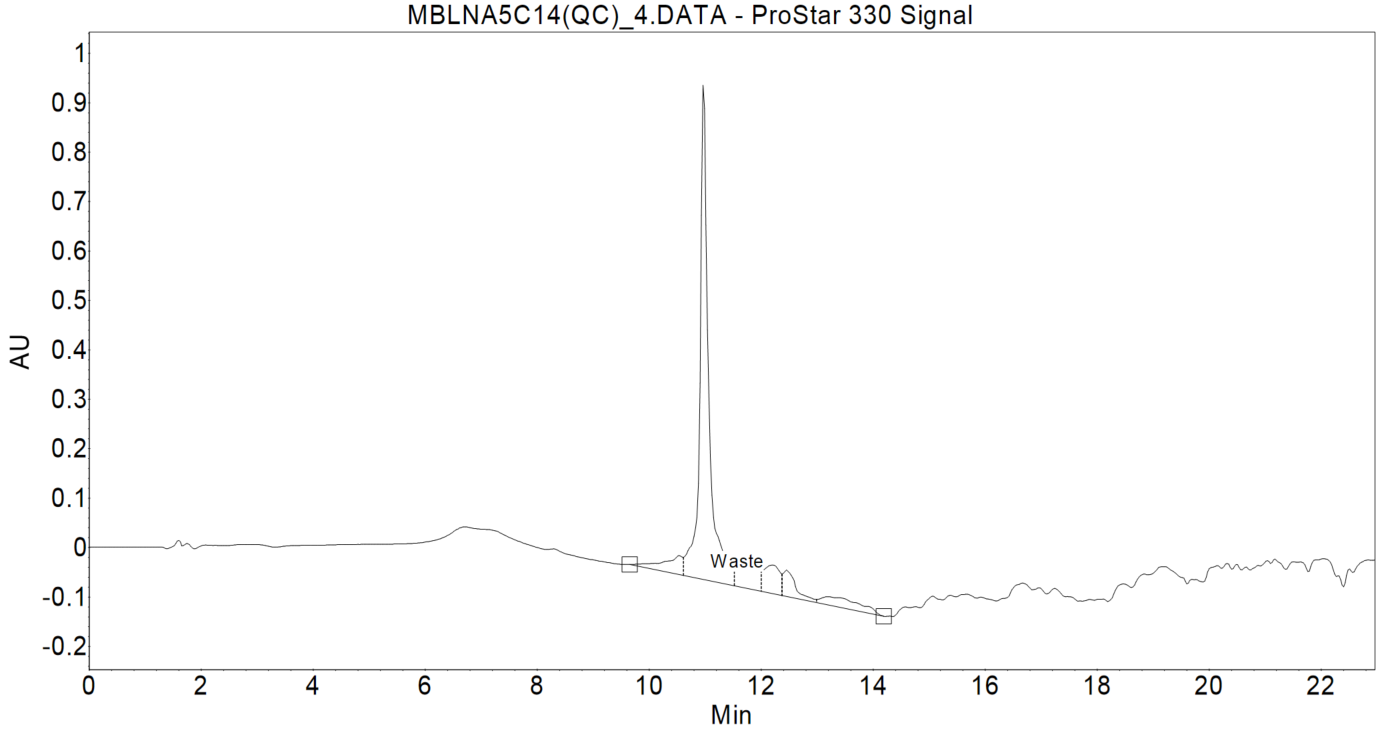

**Fig 1.** **HPLC trace (top), and unprocessed (middle) and deconvoluted (bottom) ESI-MS spectra for LNA1d.**


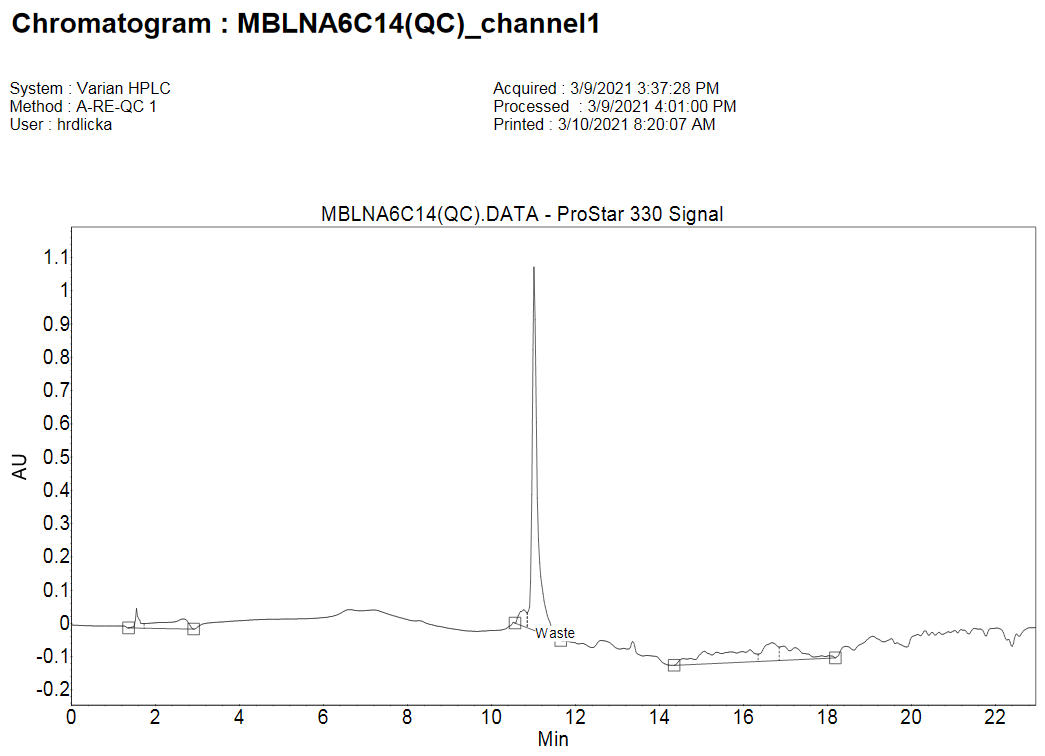


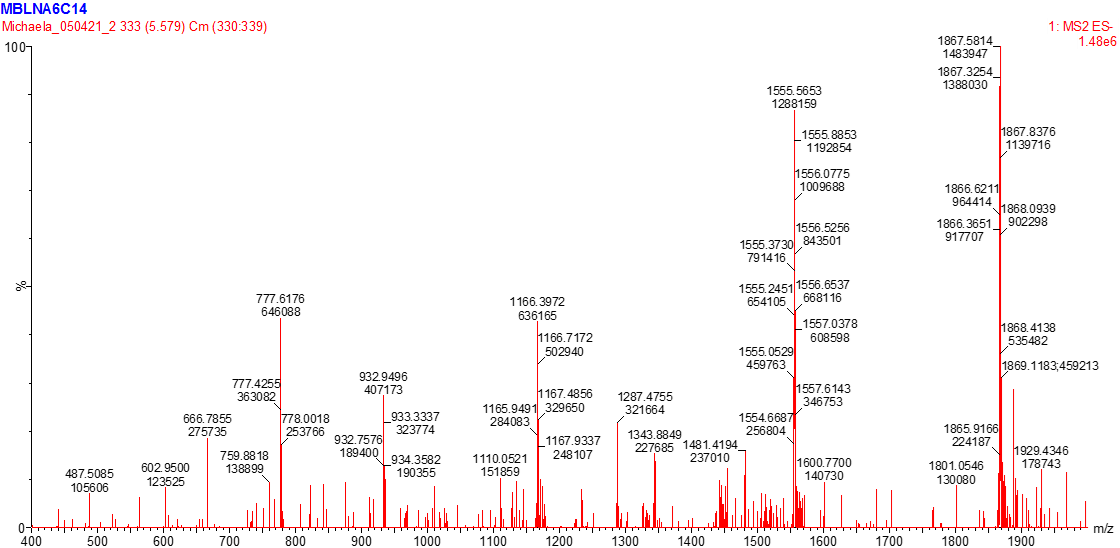


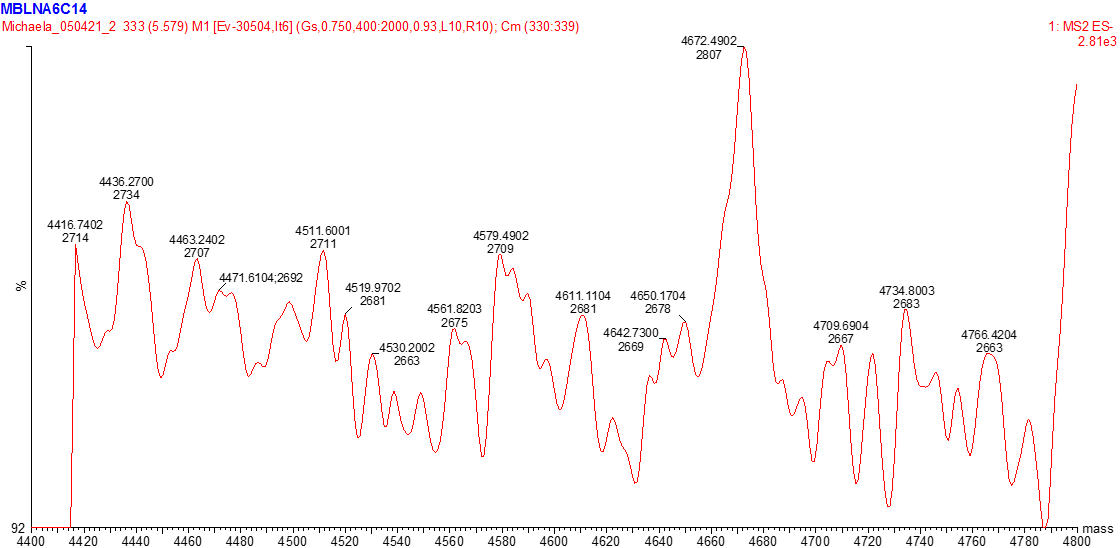


**Fig 2.** **HPLC trace (top), and unprocessed (middle) and deconvoluted (bottom) ESI-MS spectra for LNA1u.**


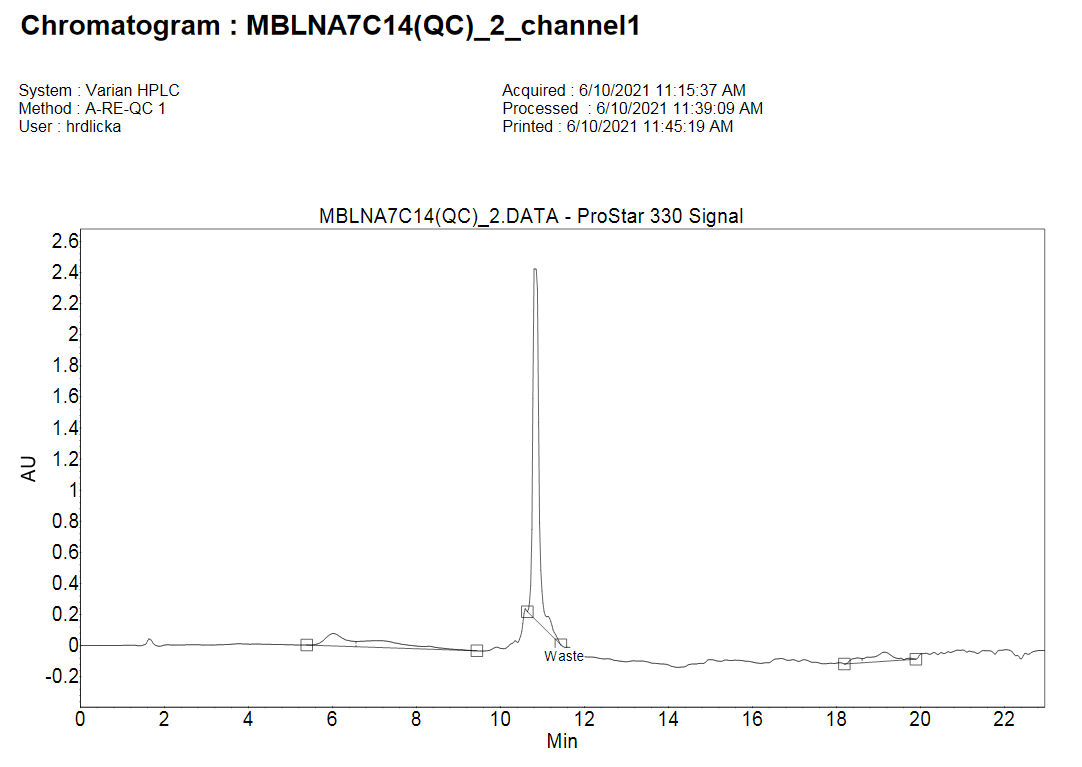


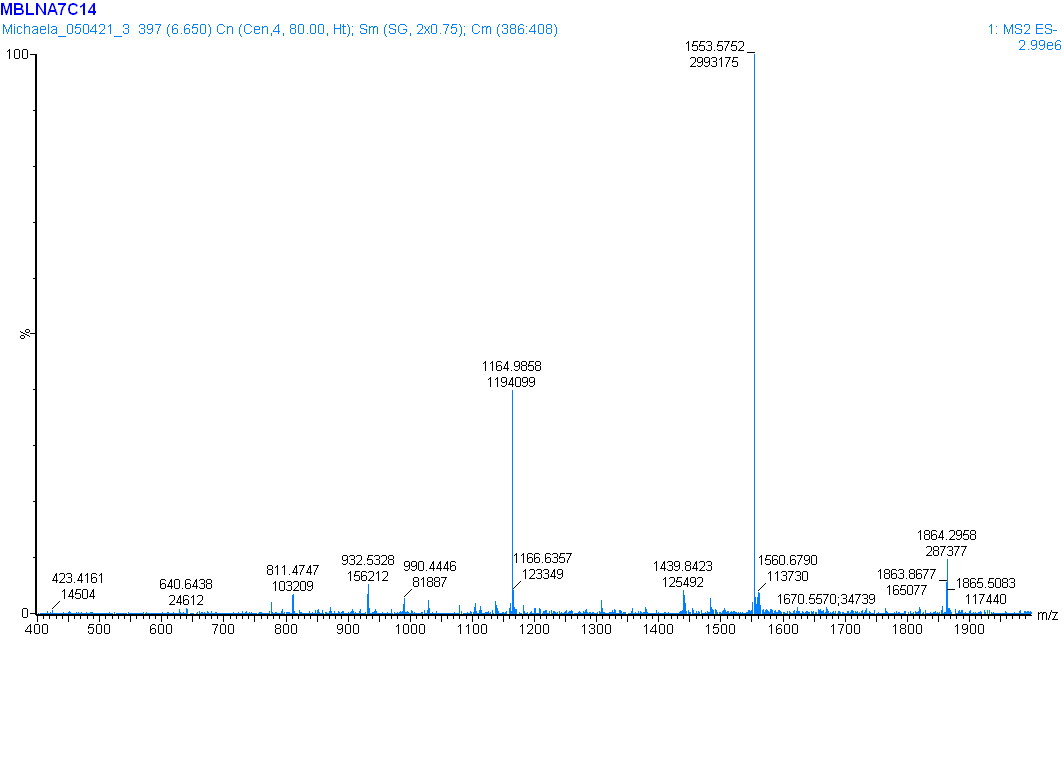

**Fig 3.** **HPLC trace (top), and unprocessed (middle) and deconvoluted (bottom) ESI-MS spectra for LNA2d.**


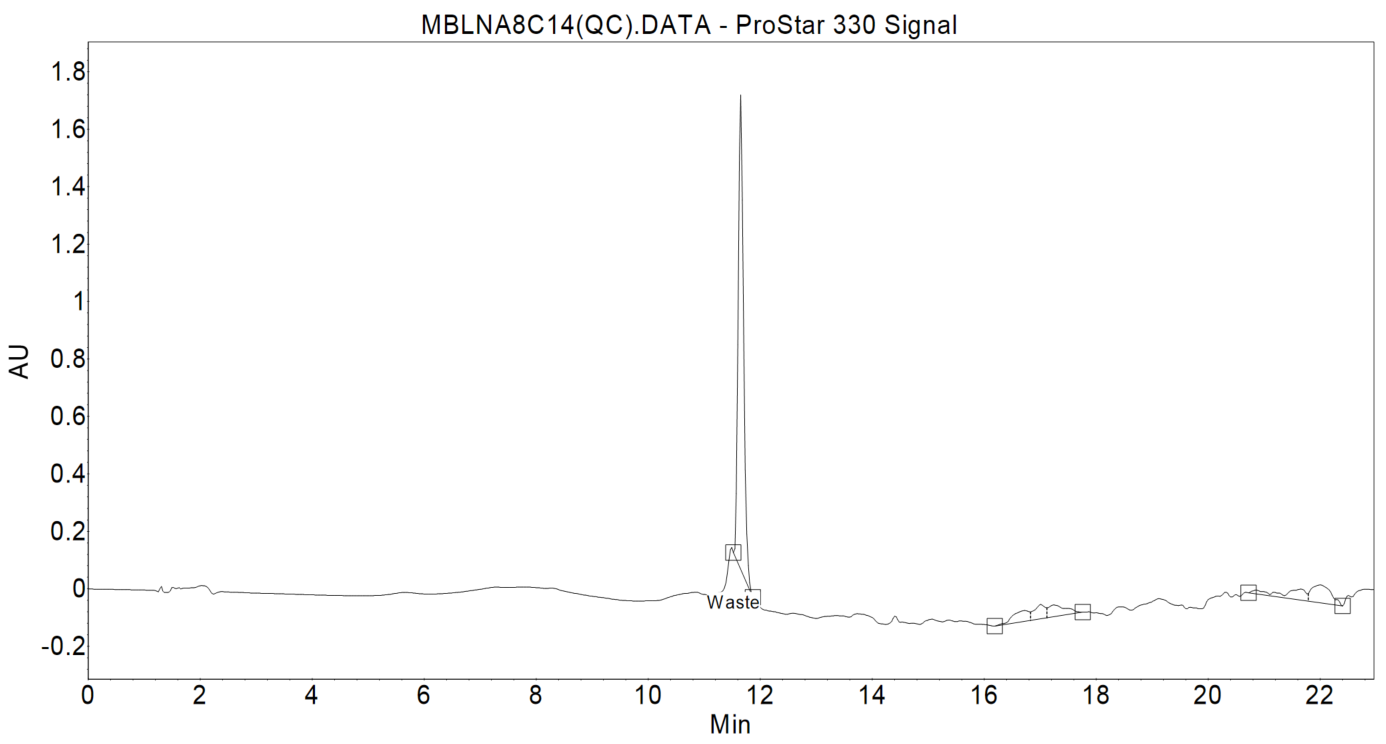


**
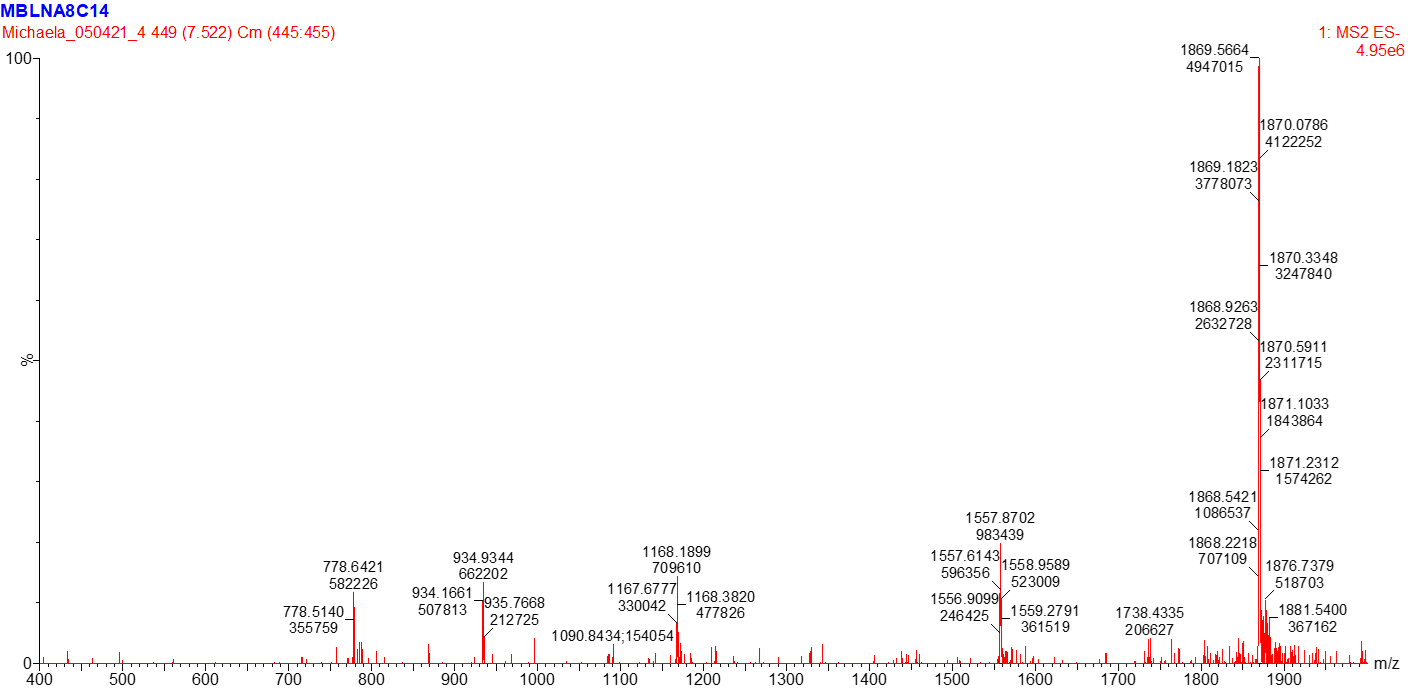
**

**
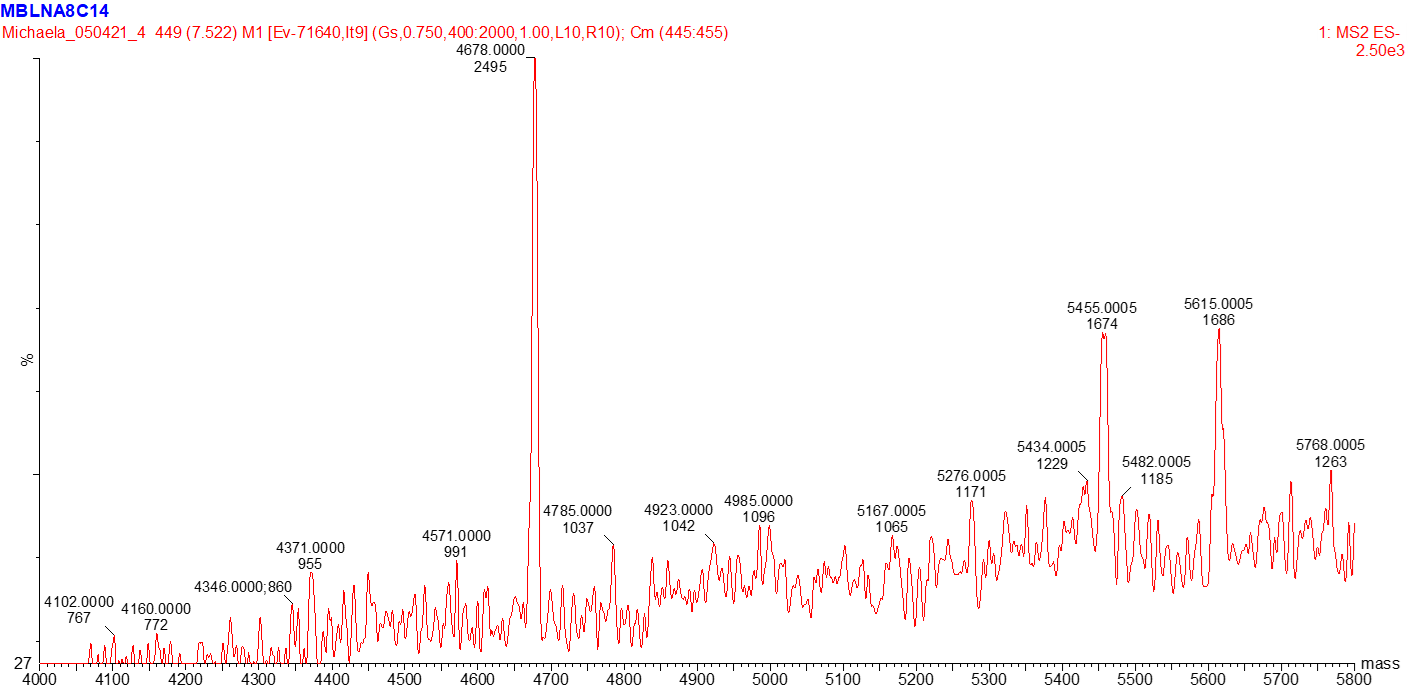
**

**Fig 4. HPLC trace (top), and unprocessed (middle) and deconvoluted (bottom) ESI-MS spectra for LNA2u.**


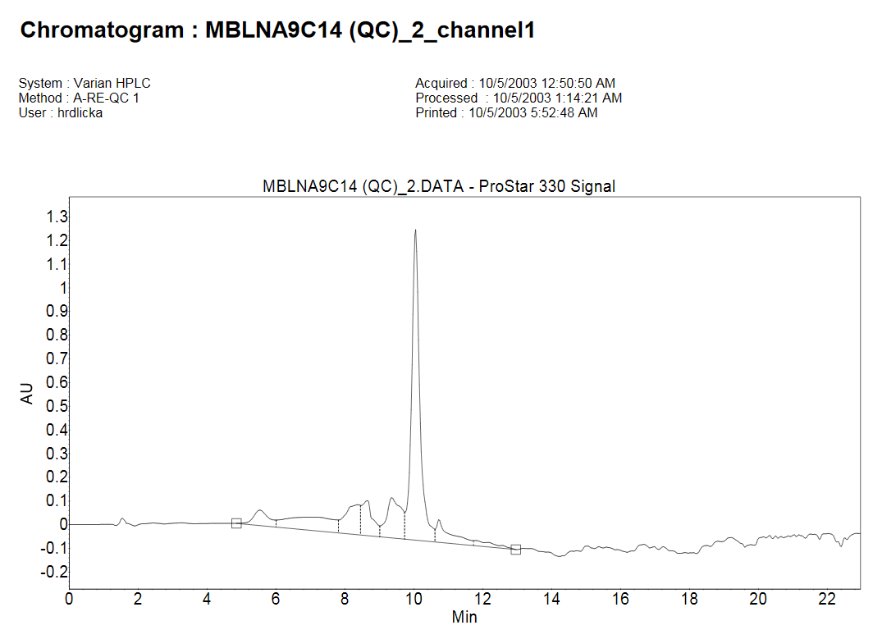


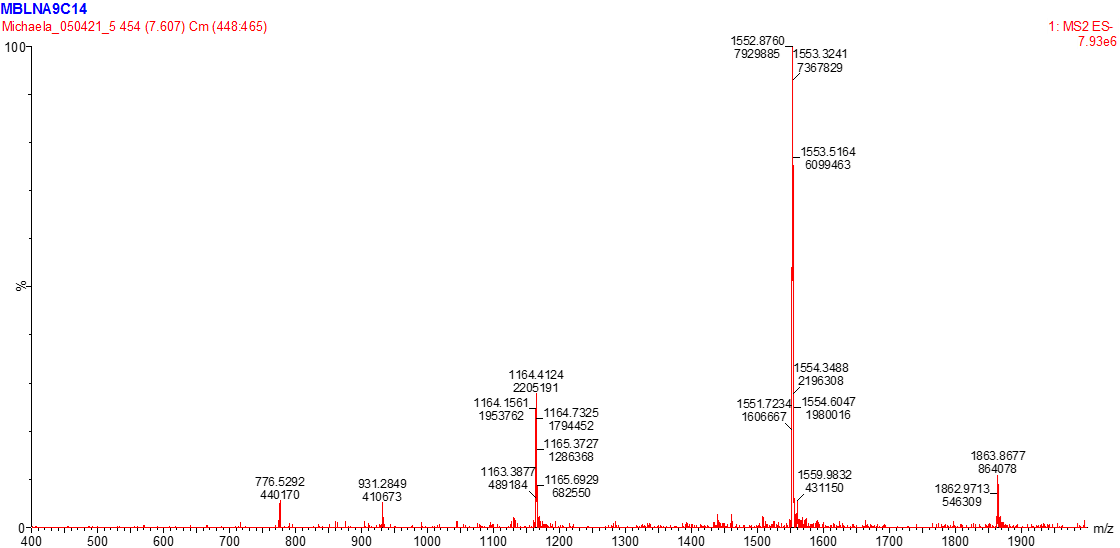


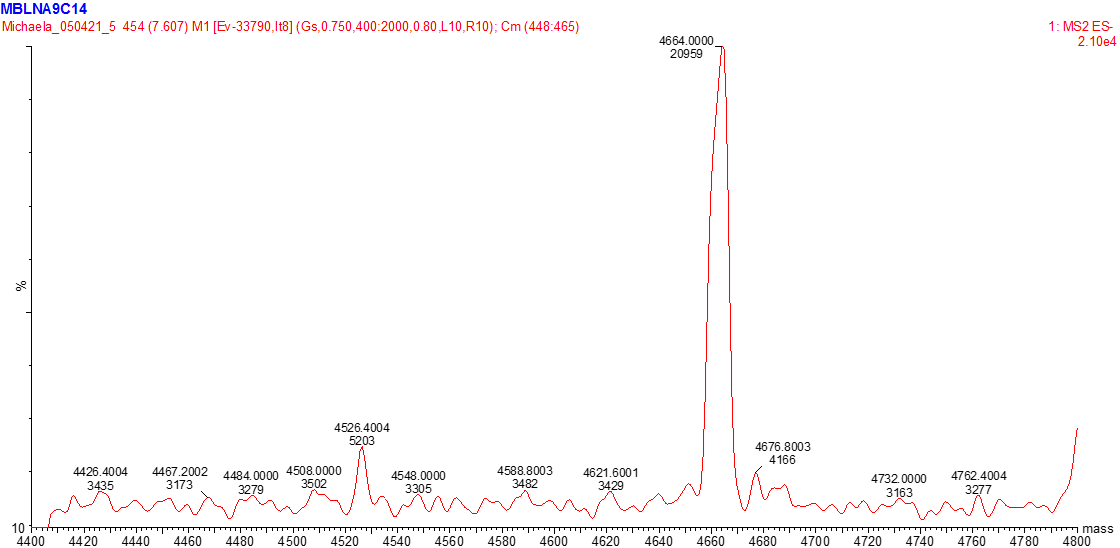


**Fig 5.** **HPLC trace (top), and unprocessed (middle) and deconvoluted (bottom) ESI-MS spectra for LNA3d.**


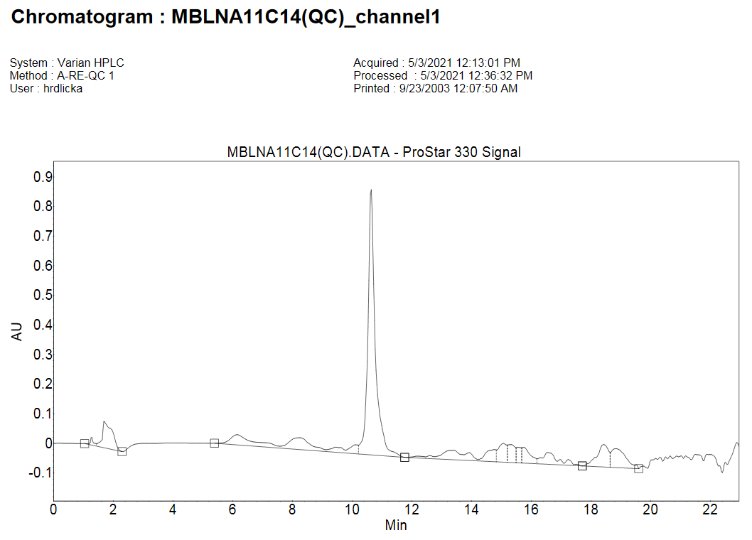


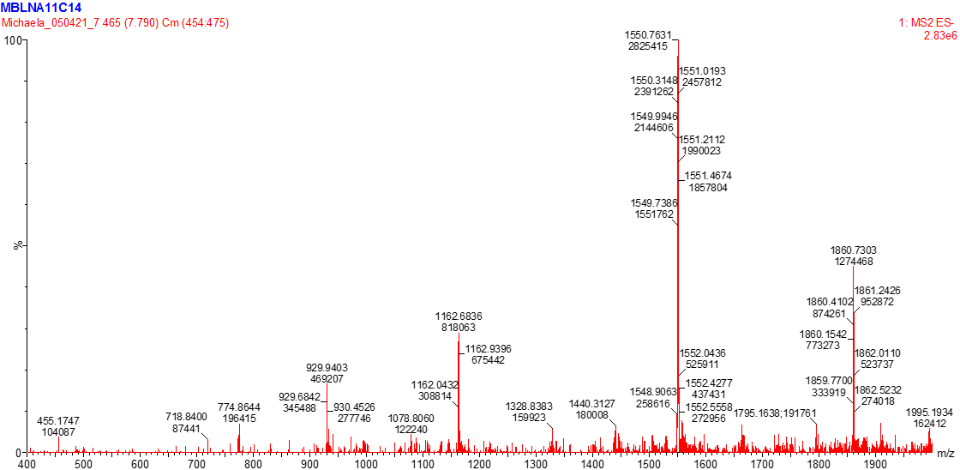


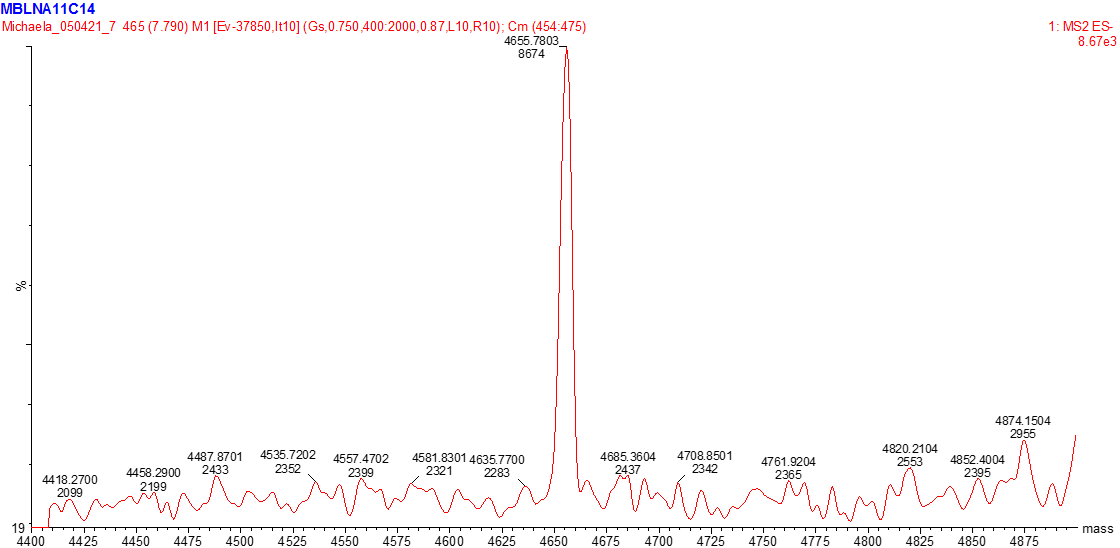


**Fig 6.** **HPLC trace (top), and unprocessed (middle) and deconvoluted (bottom) ESI-MS spectra for LNA4d.**

**Method – synthesis, purification, and characterization of LNA probes.** The LNA-modified ONs used herein (henceforth referred to as “LNAs”) were synthesized on a deoxyribonucleotide-capped long chain alkyl amine-controlled pore glass (LCAA-CPG; pore size of 500 Å) solid support using commercially available nucleobase-protected LNA monomers (Glen Research) on an automated DNA synthesizer (Expedite 8909) applying standard procedures except for extended coupling and oxidation as recommended by the manufacturer (note: the LNAs are fully modified except for a 3'-terminal DNA monomer). The LNAs were subsequently treated with 32% ammonia (55 °C, 17 h) to remove nucleobase protecting groups and cleave LNAs from the solid support. The ammonia solution was evaporated off using a centrifugal vacuum concentrator and the crude DMT-on LNAs were resuspended in 500 μL of HPLC-grade water. Detritylation and purification of LNAs was performed at room temperature using TOP-DNA 150 mg oligonucleotide cartridges (Agilent) following the manufacturer’s recommendation. Briefly described, TOP-DNA cartridges were secured in a fabricated vacuum manifold, conditioned with 0.5 mL HPLC-grade MeCN with a flow rate of ~2 drops/second, and then equilibrated with 1 mL of 2 M aqueous triethyl ammonium acetate. An aqueous NaCl solution (100 mg/mL) was added to the resuspended LNAs in equal volume (1:1 v/v) and the resulting mixture then added to the cartridge. The cartridges were rinsed with an additional 2 mL of the NaCl solution to flush out impurities. The LNAs were detritylated (2 mL, 5% aq. CF_3_COOH), rinsed with 2 mL of HPLC-grade water, and eluted from the cartridge (0.5 mL, 50:50 MeCN:H_2_O, v/v). The LNAs were dried using a centrifugal vacuum concentrator and reconstituted in HPLC-grade water. The purity and identity of the synthesized LNAs were verified using analytical HPLC (XTerra MS C_18_ column: 0.05 M TEAA and MeCN gradient; >90% Figs 2‒6) and LC-ESI-MS analysis (Waters Acquity C_18_ column; TEAA and MeCN gradient) recorded on a quadrupole time-of-flight (Q-TOF) mass spectrometer. The raw signals were deconvoluted using the Max Ent software provided with the spectrometer to obtain molecular ion peaks (Table 1 and Figs 2‒6).

**Method – thermal denaturation and UV-Vis absorption experiments.** Thermal denaturation temperatures (*T*_m_s) of single-stranded LNAs and all duplexes (1.0 μM final concentration of each strand) were recorded on a Cary 100 UV/VIS spectrophotometer, equipped with a 12-cell Peltier temperature controller and using quartz optical cells with a path length of 1.0 cm, in low salt buffer ([Na^+^] = 10 mM, pH 7.0 (NaH_2_PO_4_/Na_2_HPO_4_), [EDTA] = 0.2 mM; Figs 7 and 8, and Table 2). Samples were first annealed by heating to 90 °C (2 min) followed by cooling to the starting temperature of the experiment over 16 min. The temperature of the denaturation experiments ranged from at least 15 °C below the *T*_m_ to at least 15 °C above the *T*_m_ (although not below 10 °C nor above 95 °C). A temperature ramp of 0.5 min^−1^ was used in all experiments. *T*_m_s were taken as the maximum of the first derivative of thermal denaturation curves (*A*_260_ vs*. T*). Reported *T*_m_s are averages of at least two experiments within ± 1.0 °C. *T*_m_s for some LNA-containing duplexes were determined from differential thermal denaturation curves to eliminate the impact of secondary structures observed with certain LNA strands (i.e., differential thermal denaturation curve = differential thermal denaturation curve for LNA-containing duplex minus denaturation curve for single-stranded LNA, see below for Discussion on *T*_m_s). UV-Vis absorption spectra (range 200–600 nm) were recorded at 10 °C using *T*_m_ samples (*i.e.*, each strand used at 1.0 μM in low salt *T*_m_ buffer) (Table 3 and Fig 9).


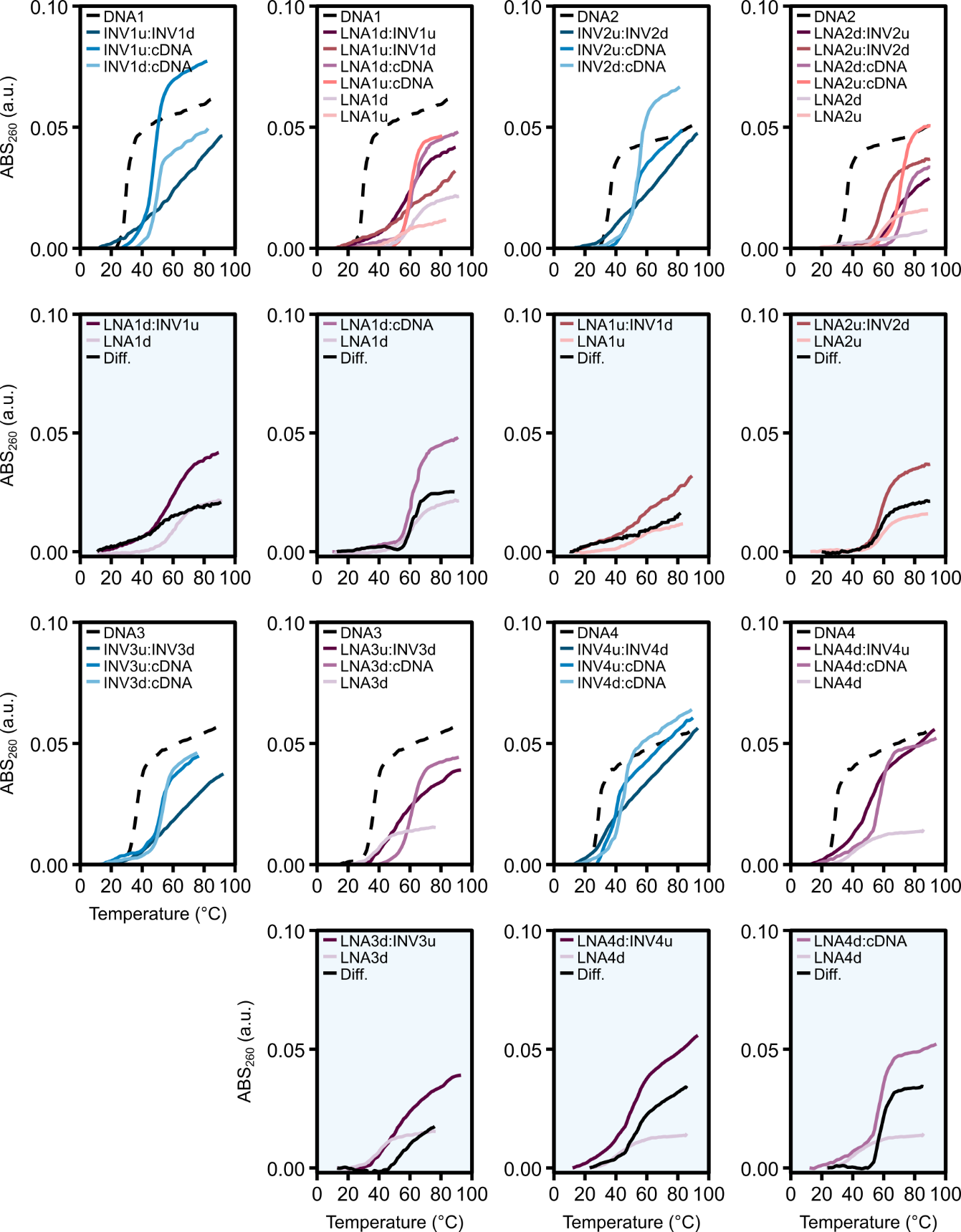


**Fig 7.** **Representative thermal denaturation profiles and differential curves for probes used herein.** Plots with white backgrounds depict raw denaturation profiles; plots with grey backgrounds depict differential (“Diff”) curves as well as the raw profiles used to construct differential curves. For sequences of strands and experimental conditions, see Table 2.

**
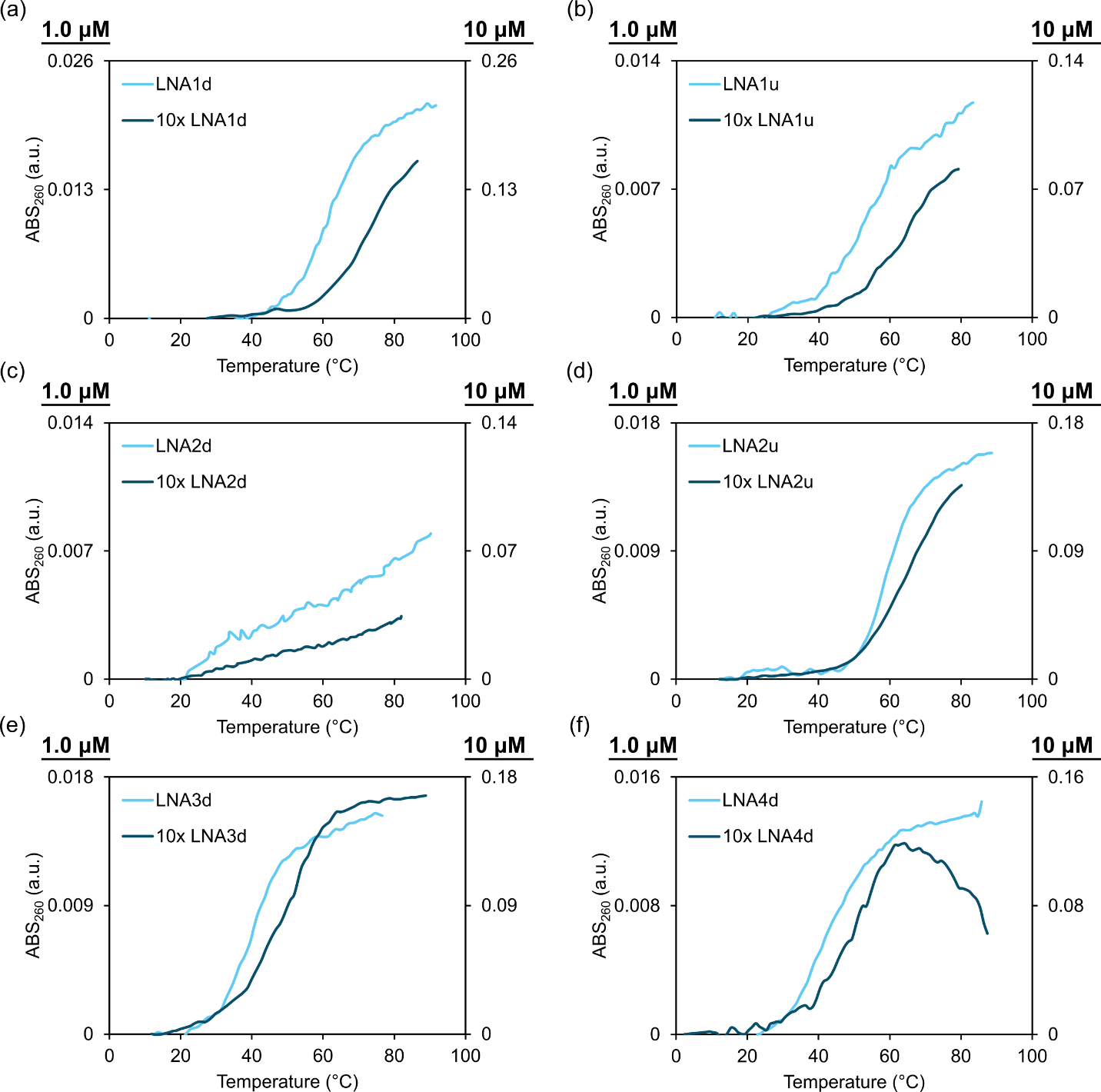
**

**Fig 8.** **Representative thermal denaturation profiles for single-stranded LNA probes.** For sequences and experimental conditions, see Table 2. Left and right Y-axes depict *A*_260_ for probes used at 1.0 µM and 10 µM concentration, respectively.

**Table 2.** **Sequences of chimeric Invader:LNA, conventional Invader, and LNA probes studied herein; *T*_m_s of double-stranded probes, duplexes between individual probe strands and cDNA, and LNA secondary structures (“ssProbe”); and TA values for double-stranded probes.** *^a^*

|  |  |  | *T*_m_ [Δ*T*_m_] (°C) | | | |  |
| --- | --- | --- | --- | --- | --- | --- | --- |
| Entry | Name | Sequence | ssProbe | dsProbe | 5'-Strand vs. cDNA | 3'-Strand vs. cDNA | TA (°C) |
| 1 | INV1u | 5'-C**U**GA**U**AGAAC**U**GTT-b | - | 51.0 *^b^* | 44.5 | 61.0 *^b^* | 28.0 |
|  | LNA1d | 3'-Gactatcttgacaa | 58.5 [+32.0] | [+24.5] | [+18.0] | [+34.5] |  |
| 2 | LNA1u | 5'-ctgatagaactgtT | 44.0 [+17.5] | nt | 58.0 | 47.0 | - |
|  | INV1d | 3'-GA**C**TA**U**CTTGA**C**AA | - |  | [+31.5] | [+20.5] |  |
| 3 | INV1u | 5'-C**U**GA**U**AGAAC**U**GTT-b | - | nt | 44.5 | 47.0 | - |
|  | INV1d | 3'-GA**C**TA**U**CTTGA**C**AA | - |  | [+18.0] | [+20.5] |  |
| 4 | INV2u | 5'-G**C**A**U**GGCTCT**U**GAT | - | 60.5 | 48.0 | 72.5 | 25.0 |
|  | LNA2d | 3'-Cgtaccgagaacta | nt | [+25.5] | [+13.0] | [+37.5] |  |
| 5 | LNA2u | 5'-gcatggctcttgaT | 55.5 [+20.5] | 56.0 *^b^* | 69.5 | 53.0 | 31.5 |
|  | INV2d | 3'-CG**U**A**C**CGAGAA**C**TA-C_6_NH_2_ | - | [+21.0] | [+34.5] | [+18.0] |  |
| 6 | INV2u | 5'-G**C**A**U**GGCTCT**U**GAT | - | ~35.0 *^c^* | 48.0 | 53.0 | ~31.0 |
|  | INV2d | 3'-CG**U**A**C**CGAGAA**C**TA-C_6_NH_2_ | - | [±0] | [+13.0] | [+18.0] |  |
| 7 | INV3u | 5'-TCGT**U**AT**U**GGC**G**AT | - | ~51.0 *^b,c^* | 50.5 | 58.5 | ~24.5 |
|  | LNA3d | 3'-Agcaataaccgcta | 37.5 [+4.0] | [+17.5] | [+17.0] | [+25.0] |  |
| 8 | INV3u | 5'-TCGT**U**AT**U**GGC**G**AT | - | nt | 50.5 | 50.5 | - |
|  | INV3d | 3'-AGCAA**U**AA**C**CGC**U**A-C_6_NH_2_ | - |  | [+17.0] | [+17.0] |  |
| 9 | INV4u | 5'-**U**A**U**GCCATT**U**GAAA | - | 55.0 *^b^* | ~41.0 *^e^* | 54.5 *^b^* | ~13.5 |
|  | LNA4d | 3'-Atacggtaaacttt | 39.5 [+12.5] | [+28.0] | [+14.0] | [+27.5] |  |
| 10 | INV4u | 5'-**U**A**U**GCCATT**U**GAAA | - | ~31.0 *^d^* | ~41.0 *^e^* | 43.0 | ~26.0 |
|  | INV4d | 3'-b-A**U**A**C**GGTAAA**C**TTT | - | [+4.0] | [+14.0] | [+16.0] |  |

*^a^* A/G/C/T indicate adenin-9-yl, guanin-9-yl, cytosin-1-yl, and thymin-1-yl DNA monomers, respectively. **U**, **C**, and **G** are 2′-*O*-(pyren-1-yl)methyluridine monomers; LNA monomers are denoted by lowercase letters (“c” = 5-methyl-cytosin-1-yl LNA monomer). Structures of modifications are shown in Fig 1 of the main manuscript. Δ*T*_m_ = change in *T*_m_ relative to the corresponding unmodified reference DNA duplex: **dsDNA1** (5'-CTGATAGAACTGTT:3'-GACTATCTTGACAA; *T*_m_ = 26.5 °C) = reference for *E. coli* (R1) probes, entries 1‒3, **dsDNA2** (5'-GCATGGCTCTTGAT:3'-CGTACCGAGAACTA; *T*_m_ = 35.0 °C) = reference for *E. coli* (R2) probes, entries 4‒6, **dsDNA3** (5'-TCGTTATUGGCGAT:3'-AGCAATAACCGCTA; *T*_m_ = 33.5 °C) = reference for *S. enterica* probes, entries 7 and 8, and **dsDNA4** (5'-TATGCCATTTGAAA:3'-ATACGGTAAACTTT; *T*_m_ = 27.0 °C) = reference for *C. jejuni* probes, entries 9 and 10. Thermal denaturation curves (Fig 7) were recorded in a low salt phosphate buffer ([Na^+^ ] = 10 mM, pH 7.0 (NaH_2_PO_4_/Na_2_HPO_4_), [EDTA] = 0.2 mM) using 1.0 µM of each strand. “nt” = no transition; “-” = not determined. *^b^ T*_m_ obtained from differential thermal denaturation curves (Fig 7). *^c^* Broad, *^d^* weak, *^e^* or irregular transition observed (Fig 7).

*T_m_ discussion.* *T*_m_s of double-stranded probes (i.e., Invader probes and chimeric Invader:LNA probes) and duplexes between individual probe strands and cDNA were determined to assess their stability (Table 2). *T*_m_s of single-stranded LNAs were also determined to assess the potential formation of secondary structures. *T*_m_s were used to calculate the thermal advantage (TA), a term we have used to estimate the driving force for recognition of complementary dsDNA targets by double-stranded probes,^1^ and which we define as TA = *T*_m_ (5'-strand vs*.* cDNA) + *T*_m_ (3'-strand vs*.* cDNA) − *T*_m_ (probe duplex) − *T*_m_ (dsDNA). More positive values indicate a more prominent driving force for dsDNA-recognition.

Consistent with our previous studies,^1,2^ most Invader probes were labile (∆*T*_m_ ~ 0‒4.0 °C) or exhibited no clear transitions (i.e., **INV1u**:**INV1d** and **INV3u**:**INV3d**), indicative of substantial duplex perturbation as the nearest neighbor exclusion principle^3,4^ is violated. In contrast, very stable duplexes were formed between individual Invader strands and cDNA (∆*T*_m_ = +13.0 to +20.5 °C), resulting in favorable TA values (between 26.0 and 31.0 °C).

Chimeric Invader:LNA probes were generally found to be surprisingly stable (∆*T*_m_ = 17.5‒28.0 °C). However, since duplexes between LNA strands and cDNA are even more stable (∆*T*_m_ = 25.0‒37.5 °C), favorable TA values are observed for chimeric probes (between ~13.5 and 31.5 °C).

Single-stranded ONs that are extensively modified with strongly affinity-enhancing modifications are often prone to formation of stable secondary structures, which may interfere with formation of duplexes with complementary DNA. *T*_m_s were therefore determined for the single-stranded LNAs (Table 2). Indeed, except for **LNA2d**, all LNA probes exhibited clear transitions (∆*T*_m_s = 4.0‒32.0 °C) indicative of stable secondary structures (Fig 7 and Table 2). Additional thermal denaturation experiments, in which the concentration of the LNA strands was increased 10-fold, were performed to discern if the observed transitions were due to inter- or intramolecular interactions (Fig 8). Transitions shifted to higher temperatures when LNA strands were used at higher concentrations, indicating the formation of homoduplexes (Fig 8). As expected, increasing the concentration of **LNA2d** had no effect on the thermal denaturation profile.

Differential thermal denaturation curves were recorded to eliminate the impact of LNA secondary structures on the *T*_m_ determination of LNA-containing duplexes (Fig 7). This was achieved by subtracting LNA-only denaturation curves from their respective LNA:INV or LNA:cDNA denaturation curves using the *Maths* function on the spectrophotometer’s accompanying software. The resulting differential curve was then used to calculate the duplex *T*_m_ (footnote b in Table 2).

*Discussion on UV-Vis characterization of the pyrene-containing oligonucleotides studied herein.* To gain insight into the placement of the pyrene moieties, UV-Vis spectra were recorded for individual Invader strands and the corresponding duplexes with complementary DNA, LNA, or Invader strands (Table 3 and Fig 9). Intercalation of the pyrene moieties upon duplex formation is expected to result in bathochromic shifts of the pyrene absorption bands relative to the individual Invader probe strands due to increased electronic interactions between pyrene moieties and nucleobases, whereas hypsochromic shifts are expected if duplex formation leads to projection of pyrene moieties into one of the duplex grooves.^5^

Individual Invader strands display pyrene absorption maxima in the 333–334 nm and 348–350 nm regions (Table 3 and Fig 9). Bathochromic shifts were observed for all Invader strands upon hybridization with cDNA, consistent with pyrene intercalation (Table 3 and Fig 9). Formation of Invader duplexes resulted in less pronounced or no bathochromic shifts, which is indicative of a distorted duplex with reduced interactions between pyrene and nucleobase moieties. Formation of chimeric Invader:LNA duplexes was generally associated with minor hypsochromic shifts, consistent with a distorted duplex and/or duplex groove placement of the pyrene moieties.

**
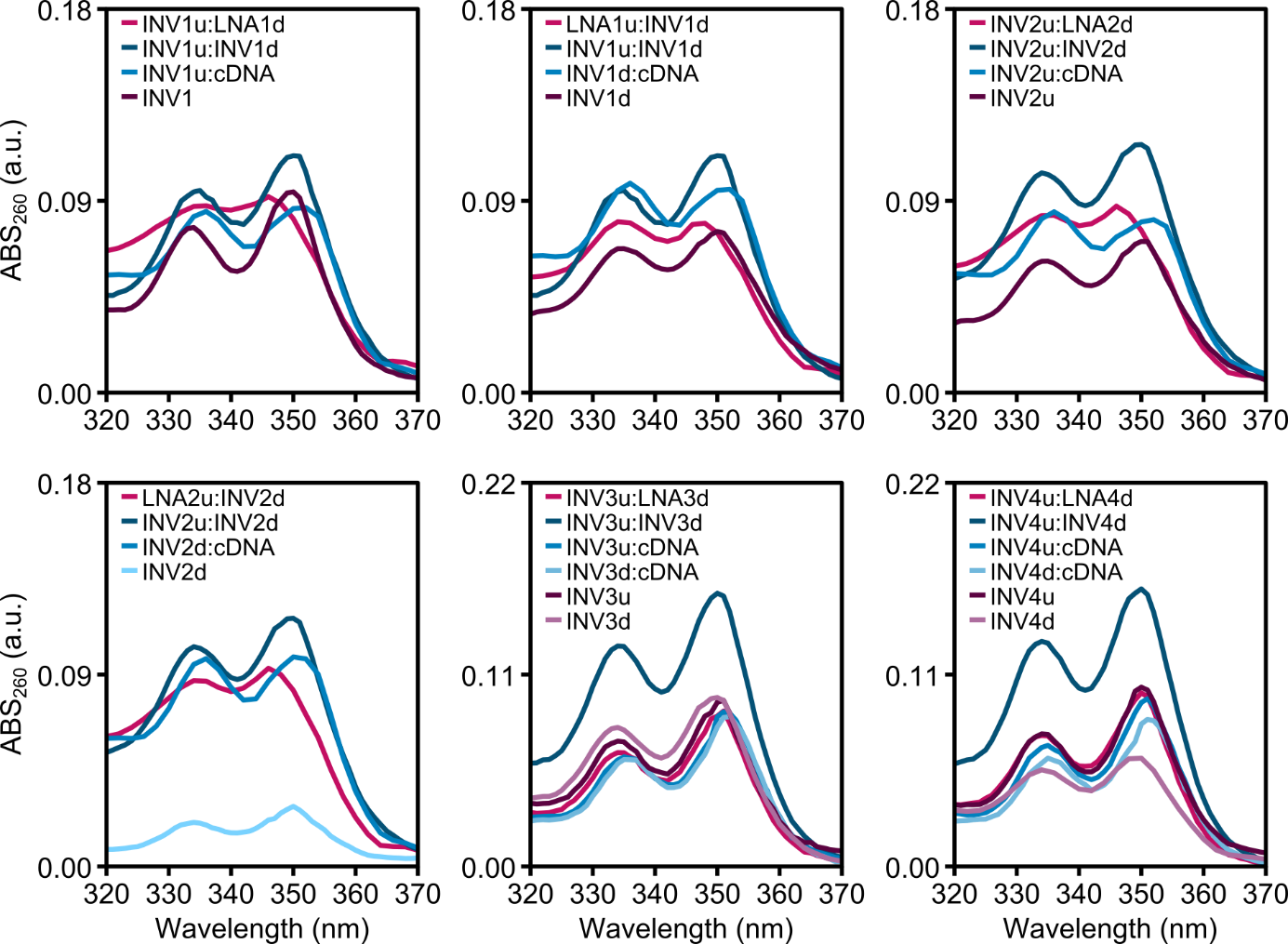
**

**Fig 9. Representative UV-Vis absorption spectra for individual Invader strands and the corresponding duplexes with complementary LNA, Invader, and DNA strands.** Spectra were recorded at 10 °C in the low salt *T*_m_ buffer using quartz optical cells with a 1.0 cm path length.

**Table 3.** **Absorption maxima in the 340–365 nm region for single-stranded Invader probes and the corresponding duplexes with complementary Invader, DNA, or LNA strands.** *^a^*

|  | | *λ*_max_ (nm) [Δ*λ*_max_] | | | |
| --- | --- | --- | --- | --- | --- |
| Probe | SSP | | +INV | +cDNA | +LNA |
| INV1u | 350 | | 350 [±0] | 352 [+2] | 348 [−2] |
| INV1d | 350 | | 350 [±0] | 352 [+2] | 348 [−2] |
| INV2u | 350 | | 350 [±0] | 353 [+3] | 348 [−2] |
| INV2d | 349 | | 350 [+1] | 352 [+3] | 348 [−1] |
| INV3u | 350 | | 350 [±0] | 351 [+1] | 351 [+1] |
| INV3d | 350 | | 350 [±0] | 352 [+2] | ND |
| INV4u | 350 | | 350 [±0] | 351 [+1] | 350 [±0] |
| INV4d | 350 | | 350 [±0] | 351 [+1] | ND |

*^a^* SSP = single-stranded probe. Δ*λ*_max_ is calculated relative to the single-stranded Invader probe. Binding partners (listed in the parenthesis) are as follows: **INV1u** (**INV1d** and **LNA1d**), **INV1d** (**INV1u** and **LNA1u**), **INV2u** (**INV2d** and **LNA2d**), **INV2d** (**INV2u** and **LNA2u**), **INV3u** (**INV3d** and **LNA3d**), **INV3d** (**INV3u**), **INV4u** (**INV4d** and **LNA4d**), and **INV4d** (**INV4u**). Spectra were recorded at 10 °C in low salt buffer using quartz optical cells with a 1.0 cm path length. ND = not determined.

**Method – control experiment evaluating linear concentration-to-signal correlation and limit of detection of assay using CDP-Star chemiluminescence substrate for the C-DiGit Blot Scanner.** Solutions of DIG-labeled DHPs, in concentrations relevant for the Invasion Assays (i.e., 5‒40 nM), were pipetted directly onto positively charged nylon membranes in 1 μL aliquots (Fig 10). DHPs were cross-linked to the membrane via UV-irradiation, processed, then imaged as described in the associated protocol. The resulting background-subtracted signal (S/N value) for each “dot” was determined by the software accompanying the C-DiGit Blot Scanner. To generate the calibration curves, S/N values were normalized to the S/N value of the highest concentration of the respective DHP (i.e., 40 nM). Normalized S/N values were averaged and plotted against the respective dose to generate the calibration curve, with error bars representing the standard deviation from three separate experiments (Fig 10a‒c). Excel’s data analysis regression tool was used to obtain the standard deviation of the y-intercept (Sy) and the slope of the calibration curve (S), which were used to calculate the limit of detection (LOD) = 3.3 × (Sy/S). The regression analysis confirms a linear correlation between concentration (in the 0‒40 nM range) and signal (R^2^ = 0.99) and estimates the LOD to be 2‒4 nM (Fig 10). Thus, the assay can be used to provide quantitative information about dsDNA-invasion by DNA-targeting probes.

**
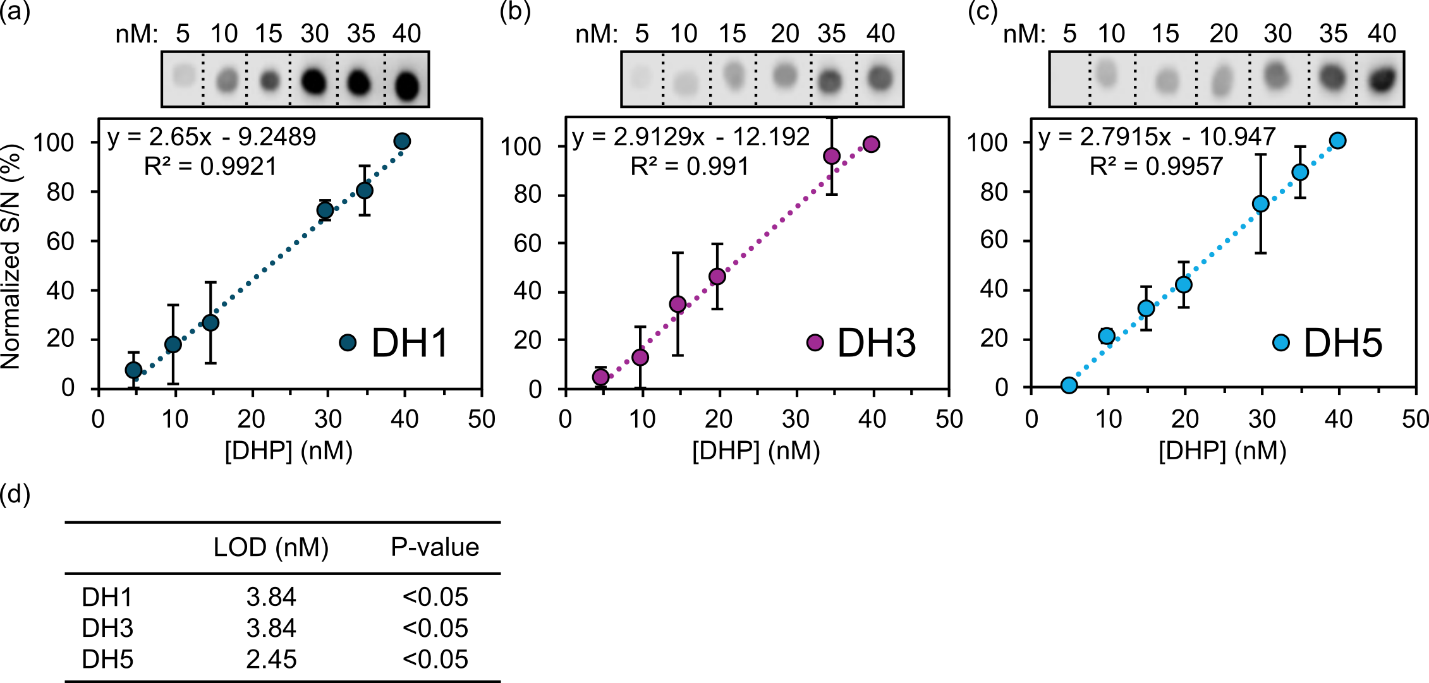
**

**Fig 10. Control experiment evaluating linear concentration-to-signal correlation and limit of detection of assay using CDP-Star chemiluminescence substrate for the C-DiGit Blot Scanner.** (a‒c) Representative dot blot images from different concentrations of the indicated DHP, and their respective calibration curves. **DH1** and **DH3** are DHPs used in this study (Fig 3 of the main manuscript), whereas **DH5** is a 13-mer DHP (5'-GGTATATATAGGC-T_10_-GCCTATATATACC-3' from a prior study.^6^

*Additional discussion on the dsDNA-targeting properties of the probes evaluated herein.* As shown in the main manuscript, the probes displayed varying levels of dsDNA-recognition (Fig 3 of the main manuscript). Invader probes and chimeric Invader:LNA probes generally resulted in substantial recognition (>50%) of the corresponding DHP targets unless the probes were exceptionally stable (see **INV2u**:**LNA2d**, *T*_m_ = 60.5 °C) and/or only moderately activated for dsDNA-recognition (see **INV2u**:**LNA2d**, **INV4u**:**LNA4d** and **INV4u**:**INV4d**, TA = 25.0 °C, ~13.5 °C and ~26.0 °C, respectively). As in our prior studies, there is no clear, consistent difference in the dsDNA-recognition characteristics of Invader probes relative to chimeric Invader:LNA probes.^6^ The more moderate levels of dsDNA-recognition displayed by the Invader and chimeric Invader:LNA probes reported herein compared to prior observations, is most likely because the Invader strands are less modified (~21.4% modified). We have previously shown that high intercalator densities increase the dsDNA-affinity of Invader probes and chimeric Invader:LNA probes.^2,6^ In addition, the use of 2′-*O*-(pyren-1-yl)methylguanosine monomers is sub-optimal^7^ (e.g., **INV3u**:**INV3d** and **INV3u**:**LNA3d**), as is the (near-)terminal placement of 2′-*O*-(pyren-1-yl)methyl-RNA monomers (e.g., **INV2u**:**LNA2d**, **INV3u**:**LNA3d**, **INV3u**:**INV3d**, **INV4u**:**LNA4d** and **INV4u**:**INV4d**).

As reported in our prior studies^1^ and observed here (Fig 3 of the main manuscript), single-stranded Invader probes display little-to-no recognition of DHP targets as both strands of an Invader probe are typically needed to ensure efficient invasion of dsDNA targets.

Some single-stranded LNA probes resulting in the formation of stable secondary structures resulted in poor dsDNA-recognition (e.g., **LNA2u**) whereas LNAs forming no or labile secondary structures resulted in prominent dsDNA-recognition (e.g., **LNA1u**, **LNA2d** and **LNA3d**). However, exceptions to these trends were also observed. For example, **LNA1d** results in prominent dsDNA-recognition despite formation of stable secondary structures, whilst **LNA4d** results in poor dsDNA-recognition despite forming a relatively labile secondary structure. These results underscore the challenges in predicting the dsDNA-targeting potential of single-stranded probes that are extensively modified with high-affinity monomers, a phenomenon that we have also previously observed with γ-modified peptide nucleic acids (PNAs).^8,9^

**References**

1. Guenther DC, Anderson GH, Karmakar S, Anderson BA, Didion BA, Guo W, et al. Invader probes: harnessing the energy of intercalation to facilitate recognition of chromosomal DNA for diagnostic applications. Chem Sci. 2015 Jul 14;6(8):5006-15. doi:10.1039/C5SC01238D
2. Shepard CP, Emehiser RG, Karmakar S, Hrdlicka PJ. Factors impacting Invader-mediated recognition of double-stranded DNA. Molecules. 2023 Jan;28(1):127. doi:10.3390/molecules28010127
3. Ihmels H, Otto D. Intercalation of organic dye molecules into double-stranded DNA—general principles and recent developments. In: Würthner F, editor. Supermolecular dye chemistry. Berlin, Heidelberg: Springer; 2005. p. 161-204. doi:10.1007/b135804
4. Persil Ö, Hud NV. Harnessing DNA intercalation. Trends Biotechnol. 2007 Oct;25(10):433-6. doi:10.1016/j.tibtech.2007.08.003
5. Asanuma H, Fujii T, Kato T, Kashida H. Coherent interactions of dyes assembled on DNA. J Photochem Photobiol C Photochem Rev. 2012 Jun;13(2):124-35. doi:10.1016/j.jphotochemrev.2012.04.002
6. Everly ME, Emehiser RG, Hrdlicka PJ. Recognition of mixed-sequence double-stranded DNA regions using chimeric Invader/LNA probes. Org Biomol Chem. 2025 Jan 15;23(3):619-28. doi:10.1039/D4OB01403K
7. Karmakar S, Guenther DC, Hrdlicka PJ. Recognition of mixed-sequence DNA duplexes: design guidelines for Invaders based on 2′-O-(pyren-1-yl)methyl-RNA monomers. J Org Chem. 2013 Dec 6;78(23):12040-8. doi:10.1021/jo402085v
8. Emehiser RG, Dhuri K, Shepard C, Karmakar S, Bahal R, Hrdlicka PJ. Serine-γPNA, Invader probes, and chimeras thereof: three probe chemistries that enable sequence-unrestricted recognition of double-stranded DNA. Org Biomol Chem. 2022 Nov 16;20(44):8714-24. doi:10.1039/D2OB01567F
9. Emehiser RG, Hrdlicka PJ. Chimeric γPNA-Invader probes: using intercalator-functionalized oligonucleotides to enhance the DNA-targeting properties of γPNA. Org Biomol Chem. 2020 Feb 21;18(7):1359-68. doi:10.1039/C9OB02726B
